# Feature library for behavioural characterization of early and late seizures in an experimental model of post-traumatic epilepsy

**DOI:** 10.1016/j.jneumeth.2025.110671

**Published:** 2026-01-03

**Authors:** Pedro Andrade, Asla Pitkänen

**Affiliations:** A. I. Virtanen Institute for Molecular Sciences, University of Eastern Finland, PO Box 1627, Kuopio FI-70211, Finland

**Keywords:** Common data elements, High resolution video-electroencephalogram, Lateral fluid-percussion, Semiology, Traumatic brain injury

## Abstract

**Background:**

To generate a behavioral feature library for a more granular description of seizure semiology in rats with traumatic brain injury (TBI). To compare the new approach to the Racine score.

**New method:**

A library of 59 seizure-related behavioral features was generated by annotating 329 seizures in 31 rats with TBI, which were monitored using high-resolution video-electroencephalogram. Of the 329 seizures, 149 were early, 85 post-electrode implantation (6th post-injury month), and 95 late seizures (7th post-injury month). Of the 59 behavioral features, 3 were pre-ictal, 43 ictal, and 13 post-ictal. Of the 43 ictal features, 7 related to consciousness, 5 to mouth and whiskers, 2 to eyes, 7 to head, 2 to ears, 6 to paws, 12 to body and tail, 2 to autonomic function, and 1 to wet-dog shakes.

**Results:**

Early, post-implantation, and late seizures showed different behavioral phenotypes (p <0.001). The number of behavioral features in post-electrode implantation and late seizures was greater than that in early seizures (p <0.05). Behavioral features did not reliably differentiate transitions from pre-ictal to ictal or from ictal to post-ictal phases.

**Comparison with existing methods:**

Ninety-one percent of early, 45 % of post-electrode implantation, and 18 % of late seizures with a Racine score of 0 showed up to 6–7 ictal-related behaviors.

**Conclusions:**

The Proposed feature list can be applied for the harmonization of data analysis and reporting, and training of video-based seizure detection algorithms to speed up non-invasive, affordable epilepsy diagnosis and assessment of treatment effects in TBI models.

## 1. Introduction

Seizure semiology comprises objective signs and subjective symptoms during epileptic seizures (Beniczky et al., 2022). These include motor, sensory, autonomic, and behavioural signs that contribute to identifying seizure onset site and progression, providing a foundation for the diagnosis and classification of seizures and epilepsy syndromes, and deciding personalized treatment (Fisher et al., 2017; Turek and Skjei, 2022). Seizure semiology is also a component of seizure severity scores used to assess the effect of new anti-seizure treatments (Pattnaik et al., 2023; Rusli et al., 2023).

Clinical characterization of seizure semiology relies on descriptions from patients, people witnessing the seizures, and video monitoring (Beniczky et al., 2022). In preclinical studies conducted in rodents, characterization of seizure semiology is largely based on on-site visual observation or retrospective analysis of video-monitored seizures either visually, or as more recently, using seizure-detection algorithms (Bertram, 2017). Seizure-associated behavioural features are typically described using the 5-stage Racine scale, with the focus on motor features, including mouth and facial clonus (stage 1), head nodding (stage 2), forelimb clonus (stage 3), rearing (stage 4), and rearing and falling (stage 5) (Racine, 1972). The Racine scale was developed to follow the progression of motor seizures in rats kindled by focal electrical stimulation of the anterior amygdala (Racine, 1972). Over the years, there have been several modifications to the original Racine scale, depending on the age of the animals or the epilepsy model investigated (Akyüz, 2020; Veliskova and Velisek, 2017a, 2017b). Still, the focus on convulsive seizures and motor features has overshadowed the detection and description of unprovoked non-convulsive seizures, recently reported to occur in several animal models (Avdic et al., 2018; Chang et al., 2018; Germonpré et al., 2020; Kharatishvili et al., 2006).

Technological advances in preclinical seizure monitoring, using cage-specific high-resolution video cameras and the application of machine learning in video signal analysis, allow recording of subtle behavioural ictal and interictal features in rodents (Gschwind et al., 2023; Hsu and Yttri, 2021; Luxem et al., 2022; Mathis et al., 2018; Mullen et al., 2024; Sabnis et al., 2024; Weinreb et al., 2024; Yu et al., 2025). So far, the studies focusing on ictal features have investigated seizures induced with pentylenetetrazol, pilocarpine, or electrical stimulation using Racine scoring as a reference (Gschwind et al., 2023; Mullen et al., 2024; Sabnis et al., 2024; Yu et al., 2025). As the behavioural seizure phenotypes vary between the epilepsy models and even in a given animal, the automated video-analysis algorithms need model-specific tailoring to optimize performance in the detection of the whole spectrum of seizure-related behavioural features in animal cohorts, including pre-ictal, ictal, and post-ictal phases at different periods of the epileptogenic process.

Traumatic brain injury (TBI) comprises about 10–15 % of structural epilepsies in humans (Herman, 2002). TBI triggers epileptogenesis also in rats and mice, leading to post-traumatic epilepsy (PTE) (Dulla and Pitkänen, 2021). So far, most of the studies performed in rat and mouse models of PTE have focused on late seizures and described their behavioural features using the Racine scale (Bolkvadze and Pitkänen, 2012; Campbell et al., 2014; Kharatishvili et al., 2006; Miszczuk et al., 2016; Shultz et al., 2013a, 2013b; Statler et al., 2009; Wang et al., 2016; Zhu et al., 2019). However, studies recording EEG right after TBI or sham operation have demonstrated the occurrence of convulsive and non-convulsive seizures in injured and sham-operated animals, and even in naïve electrode-implanted animals during the first post-operative days (Andrade et al., 2019; Bugay et al., 2020; Guo et al., 2013; Kharatishvili et al., 2006; Liu et al., 2016; Wang et al., 2017). These early or electrode-implantation-related seizures have remained largely unreported, as the EEG recordings are often started about 1 week after electrode implantation to allow the animals to recover from the surgery-related stress. The other challenge relates to substantially lower frequency of late seizures in rodents with PTE as compared to, for example, rodents developing epilepsy after status epilepticus, requiring laborious and extensive long-term video-electroencephalogram (vEEG) monitoring. Therefore, novel, affordable, non-invasive seizure detection approaches are needed to speed up epilepsy diagnosis and assessment of treatment effects after experimental TBI.

Our objective was to provide a detailed description of the semiology of seizures occurring at acute and chronic phases of TBI. We hypothesized that seizure semiology will gain more complex features over time. Rats were monitored using vEEG, including video monitoring with cage-specific high-resolution cameras. Based on behavioural annotation of 329 seizures, we generated a feature library, including 59 behaviours occurring before, during, or after electrographic seizures. The feature library developed can be integrated into the available common data elements (CDEs) (Barker-Haliski et al., 2022) and used for more accurate training of machine-learning algorithms for non-invasive video-based seizure detection after TBI and other aetiologies.

## 2. Materials and methods

**Fig. 1.**
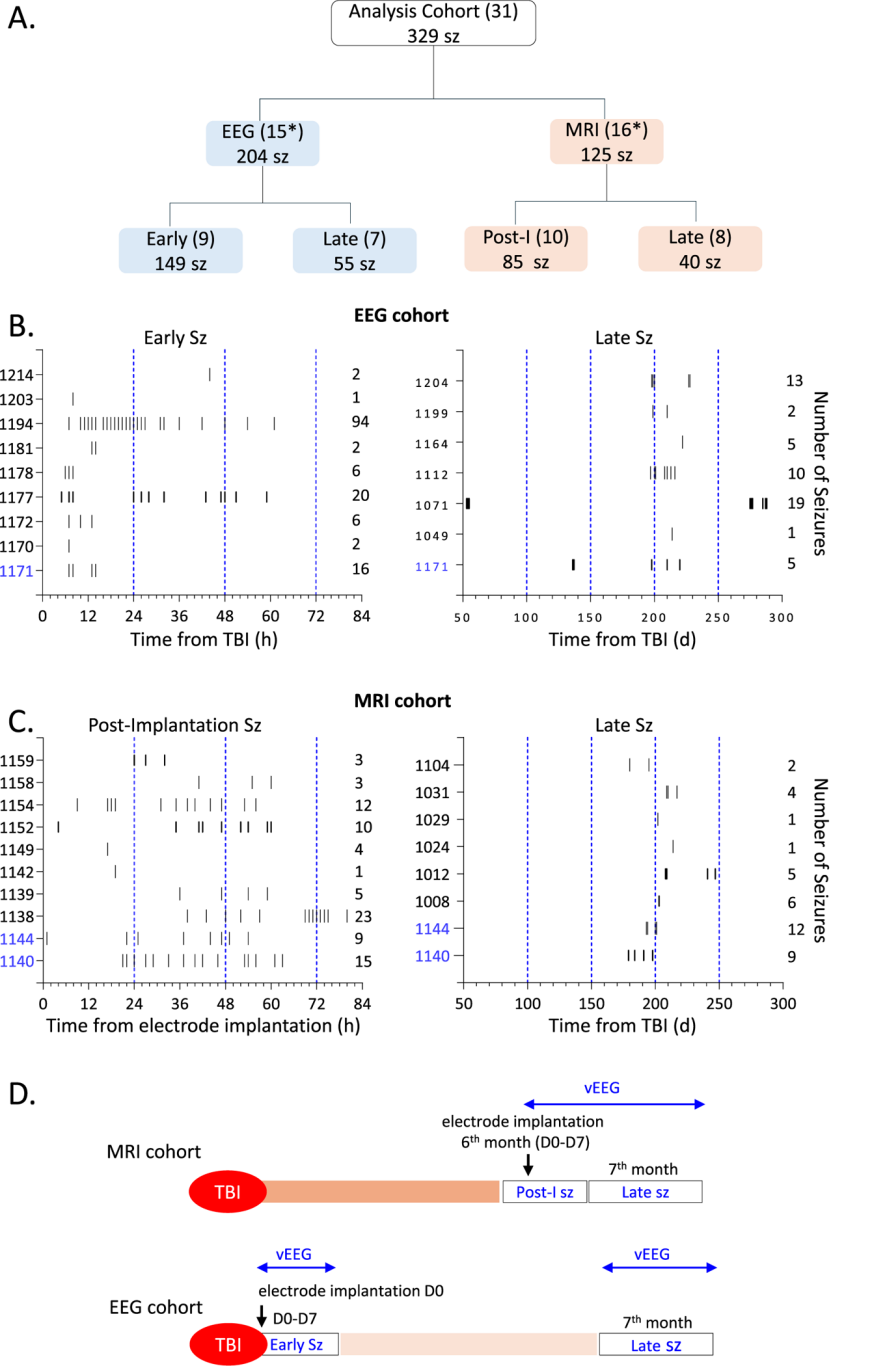
Analysis cohort and study design. **(A)** Number of early, post-implantation, and late seizures analyzed in the EEG and/or MRI cohorts. The number of animals is in parentheses. A total of 329 seizures detected in 31 rats were analyzed. Of these, 149 were early seizures (D0-D7), 85 post-implantation seizures, and 95 late seizures (55 in the EEG and 40 in the MRI cohort). Note that both early and late seizures were analyzed from 1 rat (EEG cohort) and both post-implantation and late seizures (MRI cohort) from 2 rats (asterisk). **(B)** Raster plot showing the occurrence of early and late (7th post-TBI month) seizures in the EEG cohort. Note that most of the early seizures occurred within 72 h. Both early and late seizures were analyzed in one rat (#1171, blue color). **(C)** Occurrence of post-implantation (D0-D7 after electrode implantation during the 6th post-TBI month) and late seizures in the MRI cohort. Note that most of the post-implantation seizures occurred within 72 h. Both post-implantation and late seizures were analyzed in two rats (#1140; #1144, blue color). In B and C, each tick mark represents one seizure. **(D)** In the MRI cohort, electrodes were implanted during the 6th post-TBI month and continuous 24/7 video-EEG was started immediately. Only the seizures occurring *>* 7 days after electrode implantation on the 7th post-injury month were considered late seizures (diagnostic for post-traumatic epilepsy). Seizures occurring during the first 7 post-implantation days were considered “electrode-implantation related seizures.” In the EEG cohort, electrodes were implanted right after induction of the impact. Then, video-EEG monitoring was started and continued 24/7 for the first week, and thereafter for 1 week every month. On the 7th month, monitoring was continuous for 30 days. ***Abbreviations:*** D, day; EEG, electroencephalogram; FPI, fluid-percussion injury; h, hour; MRI, magnetic resonance imaging; Post-I, post-implantation; sz, seizure; TBI, traumatic brain injury; TBI-, TBI rats without epilepsy; TBI+, TBI rats with epilepsy.

The analysis cohort, seizure occurrence, and study design are summarised in Fig. 1. The animals included in the present analysis were generated at the University of Eastern Finland (UEF) for the National Institute of Health-funded Centers-without-Walls Epilepsy Bioinformatics study (EpiBioS4Rx) (https://epibios.loni.usc.edu/; Ndode-Ekane et al., 2024). The UEF study cohort comprised two animal sub-cohorts: the MRI cohort (14 sham, 84 TBI) and the EEG cohort (total 16 sham, 70 TBI) that underwent the epilepsy phenotyping using a 1-month vEEG during the 7th month (Fig. 1D).

The present analysis cohort is a sub-cohort of the UEF EpiBioS4Rx cohort, including 31 rats with TBI. Of the 31 rats, 16 belonged to the MRI cohort and were used to analyze post-electrode implantation [day (D) 0-D7 at the end of the 6th month] and late (7th month) seizures (Fig. 1A). Fifteen rats belonged to the EEG cohort and were used to analyze early (D0-D7 after TBI) and late (7th month) seizures (Fig. 1A). In addition to the availability of a defined epilepsy phenotype (TBI-, no epilepsy; TBI+, epilepsy), the selection criteria of rats into the present analysis included (a) acceptable quality of the early, post-implantation, or late EEGs and (b) availability of a high-quality video recording of the electrographic seizure.

### 2.1. Animals and housing conditions

Adult male Sprague Dawley rats (300 – 350 g at the time of injury) were used. Animals were quarantined for 1 week (3 – 6 per cage) upon arrival at the animal facility. Thereafter, they were individually housed in a controlled environment (temperature 22 ± 1^◦^C; humidity 50 %–60 %; lights on from 07:00–19:00 h) until the end of the experiments. Food and water were provided *ad libitum* for the duration of the study. All animal procedures were approved by the Animal Ethics Committee of the Provincial Government of The Southern Finland (license number ESAVI/5146/04.10.07/2014) and carried out in accordance with the guidelines of the European Community Council Directives 2010/63/EU.

### 2.2. Induction of lateral fluid-percussion injury (FPI)

Severe TBI was induced by lateral FPI as previously described (Ndode-Ekane et al., 2024). Briefly, the rat was placed into an anaesthesia induction chamber, and isoflurane anaesthesia was induced at 5 % (room air as carrier gas; Somnosuite, # SS6069B, Kent Scientific, Torrington, CT, USA). Then, an anesthetized rat was mounted in a stereotaxic frame, a probe was inserted into the rectum to continuously assess core temperature, and a heating pad was placed below the abdomen. The temperature of the heating pad was regulated based on the animal’s core temperature (max 38 ^◦^C). Isoflurane was delivered via a nose cone mounted on the stereotaxic frame and maintained at 2 % throughout the surgery. The scalp incision site was shaved and cleaned using sterile 0.9 % NaCl before subcutaneous injection with 0.5 % lidocaine (7 mg/kg). Approximately 3–5 min later, a midline incision was made, and the surface of the skull was cleaned. A craniotomy, 5-mm in diameter (center coordinate: anteroposterior [AP] − 4.5 mm from bregma; mediolateral [ML] 2.5 mm) was made over the left cortex using a handheld trephine with the dura left intact. A plastic female Luer-lock connector was inserted into the craniotomy vertical to the skull surface, and its edges were sealed with tissue adhesive glue (3 M VetbondTM, 3 M Deutschland GmbH Germany). Dental acrylate was spread around the Luer-lock, and the connector setup was secured to the skull with an ipsilateral frontal screw. To induce TBI, the Luer-lock was filled with saline, and the rat was connected to a straight-tipped fluid-percussion device (Model FP 302, AmScien Instruments, Richmond, VA, USA). The pressure was adjusted to produce severe injury with an anticipated mortality rate of 20–30 % within the first 48 h. The mean impact pressure was 2.9 ± 0.01 atm (range: 2.4–3.3 atm). The duration of the impact was < 1 s. After injury, the rat was disconnected from the device, placed on a heating pad, and a rectal temperature probe was inserted. Occurrence of post-impact seizure-like behaviour and its duration, duration of the post-impact apnoea, and time to return of the righting reflex were recorded. To reduce the impact of experimenter-induced variability in the experimental outcome, all surgical procedures, including TBI induction and electrode implantations, were performed by the same person.

### 2.3. Electrode implantation

#### EEG Cohort.

The placement of electrodes was performed immediately after the lateral FPI. After the rat regained the righting reflex, it was re-anesthetized with 5 % isoflurane and positioned in a stereotaxic frame. Four epidural stainless-steel screw electrodes (EM12/20/SPC; P1 Technologies, Roanoke, VA, USA) were surgically implanted into the skull, according to coordinates of the rat brain atlas of Paxinos and Watson (2007). Two epidural screw electrodes were placed ipsilaterally in the frontal cortex (C3, AP: − 1.7, ML: left 2.5) and parieto-occipital cortex (O1, AP: − 7.6, ML: left 2.5). The other two screw electrodes were placed contralaterally in the frontal cortex (C4, AP: right 1.7; ML: − 2.5) and parieto-occipital cortex (O2, AP: − 7.6; ML: right 2.5). Additionally, three intracerebral tungsten bipolar recording electrodes (EM12/3–2TW/SPC; P1 Technologies; Ø 0.5 mm, tip separation 1.0 mm) were implanted ipsilaterally in the anterior perilesional cortex (AP: − 1.72; ML: − 4.0; DV: 1.8), septal hippocampus (AP: − 3.0; ML: − 1.4; DV: 3.6), and posterior perilesional cortex (AP: − 7.56; ML: − 4.0; DV: 1.8). An epidural screw electrode was placed ipsilaterally posterior to lambda as a ground, while another screw electrode served as a reference electrode and was placed contralaterally.

#### MRI Cohort.

In the MRI cohort, electrode implantation took place approximately 6 months after TBI. The locations of the epidural recording screws, ground, and reference electrodes were identical to those used in the EEG cohort. However, due to progressive brain atrophy and ventricle enlargement over time, compromising the accuracy of depth electrode placements, we first assessed the extent of cortical and hippocampal atrophy in each rat using T2-weighted MRI on D150 (see details in Ndode-Ekane et al., 2022). Based on this analysis, the planned target atlas coordinates were adjusted to position the electrodes to the same locations as in the EEG cohort. Also, the distance between the tips of the bipolar electrodes was reduced from 1.0 mm to 0.5 mm to accommodate the atrophied cortex (upper tip in layer I, lower tip in layer V) or hippocampus (upper tip in CA1, lower tip in hilus).

### 2.4. Video-EEG monitoring

#### EEG.

Following electrode implantation, each rat was placed in a specially designed Plexiglas EEG recording chamber (29 cm wide, 44 cm long, and 50 cm high, accommodating one rat per chamber) for long-term monitoring (24/7). The electrode headset was connected to a 12-pin swivel commutator (SL12C, PlasticsOne Inc.) through a flexible shielded cable (M12C-363/2, PlasticsOne Inc.), enabling free movement during EEG recordings. The commutator was then connected to an amplifier via a flexible shielded cable (363/2–441/12, PlasticsOne Inc.). High-definition electrical brain activity was recorded using a 320-channel Digital Lynx 16SX amplifier (Neuralynx, USA) with a sampling rate of 5 kHz. The amplifier had an analog bandwidth ranging from DC to 40 kHz and offered 8 independent analog references for every 32 channels, allowing customization for each animal. Individual channel data underwent 24-bit conversion.

#### Video.

Monitoring of each animal occurred through a single high-resolution camera (Basler acA1300–75gm GigE, Basler, Germany) configured to capture 30 frames per second (fps) with a resolution of 1.3 megapixels. The video was compressed using H.264. Infrared illumination specific to each cage (24 V, 150 mA) was utilized for night-time recordings. The synchronization of EEG and video data was achieved with nanosecond precision using the precision time protocol IEEE-1588. Particular attention was paid to align the camera with the cage to capture a focused video of the entire animal while it moved in the cage.

The vEEG system generated approximately 1.5 TB of data within a 24-hour period. For data storage, the video-EEG system was connected to a network-attached storage system consisting of 500 TB of storage configured with RAID6 for data redundancy. For analysis, each vEEG file was imported into Spike2 (version 9, CED, UK) and analysed visually in 30-s epochs on a display.

### 2.5. EEG and video analysis

#### 2.5.1. Detection of electrographic seizures

##### Early seizures (D0-D7 after TBI) and post-implantation seizures (6th month, D0-D7 after electrode implantation).

EEGs were screened visually on the computer screen, and seizures were annotated by an experienced technician. Then, two investigators (P.A. and A.P.) screened all annotated seizures in vEEG, and noted their sleep-wake cycle occurrence, onset electrode, propagation pattern, and duration.

##### Late seizures.

First, seizures were annotated in the 1-month-long EEG using the automated seizure detection algorithm that was designed for late seizure detection (Andrade et al., 2018). Then, an experienced technician visually confirmed all seizure candidates on a computer screen. Finally, two investigators (P.A. and A.P.) screened all seizures in video-EEG and noted their sleep-wake cycle occurrence, onset electrode, propagation pattern, and duration.

### 2.5.2. Analysis of behavioural features of electrographic seizures on high-resolution videos

Our initial behavioural scoring of early and late electrographic seizures using the Racine scale turned out to apply to only a small proportion of seizures (Racine, 1972). Therefore, we first generated a feature list that included the features related to seizure onset, ictal phase, and post-ictal phase. The features were defined in videos that had been time-locked with EEG (Table 1, examples in Video 1). Then, for each seizure, the temporal evolution of behaviours was annotated from b_1_ (first behaviour) to b_x_ (last behaviour). Examples of the annotation process are presented in Videos 1–4. In each seizure, a given feature was annotated only once, even though the animal could express several episodes of that feature during a seizure (e.g., more than one period of immobility or wet-dog shakes).

For comparison, we also scored the seizures using the Racine scale (Racine, 1972). Score 1- mouth and face clonus, head nodding; Score 2 – clonic jerks of one forelimb, Score 3 – bilateral forelimb clonus, Score 4 – forelimb clonus and rearing, Score 5 – forelimb clonus with earing and falling. In addition, we had Score 0 – no behaviours matching with Racine scale.

#### 2.5.3. Analysis of pre-ictal and post-ictal states

##### Pre-ictal behaviour.

Pre-ictal phase was defined as the preceding 5 s of electrographic seizure onset. The last pre-ictal feature was classified as awake, sleep, or immobility. Justification of “awake” or “sleep” state was based on EEG analysis, in which we annotated wake, N2, N3, and rapid-eye-movement (REM) sleep (Andrade et al., 2022). Pre-ictal behaviour was considered “immobility” if the sleep-wake state in an immobile animal could not be defined due to a pathological EEG. Immobility was a typical behaviour during the first 3 days after TBI and in animals with seizure clusters during the 6th or 7th month.

##### Post-ictal behaviour.

The post-ictal behaviours were annotated during the 30 s immediately following the end of the electrographic seizure, based on video analysis.

### 2.6. Statistics

Data were analysed using GraphPad Prism v.10.4.0 and SPSS 29.0.2.0. Differences in the duration of anaesthesia between the rats in the MRI and EEG cohorts, as well as differences in the number of features and the duration of seizures between the seizure categories (early, post-implantation, and late seizures), were calculated using the Mann-Whitney *U* test. Differences in the occurrence of features in different seizure categories were analysed using the χ^2^ test. Data are shown as mean ± standard deviation of the mean. A p-value < 0.05 was considered significant.

## 3. Results

### 3.1. Materials analysed

#### 3.1.1. Analysis cohort

The 31 rats in the analysis cohort expressed a total of 329 seizures (204 seizures in 15 rats in the EEG cohort; 125 seizures in 16 rats in the MRI cohort) with high-quality EEG and video (Fig. 1A). Of these, 149 were early (9 rats), 85 post-implantation (10 rats), and 95 late (15 rats) seizures.

#### 3.1.2. Seizure occurrence relative to injury and epileptogenesis

It is important to note that the early, post-implantation, and late seizures occurred in different contexts relating to epileptogenesis and evolution of brain pathology.

Early seizures occurred after acute brain injury, typically after recovery from isoflurane anaesthesia during the 5–61 h post-impact (Fig. 1B – left panel). Due to brain damage, analysis of sleep in the EEG and presumed conscious behaviours in the video were compromised.

The post-implantation seizures were monitored about 5.5 months (160 d) after TBI. They occurred mostly within 42 h (median 43.5, range 0–80) after induction of isoflurane anaesthesia and implantation of epidural and depth electrodes (Fig. 1C – left panel). Importantly, by that time, the animal had recovered from the injury, and sleep/wake epochs could be identified in the EEG for most of the time. Also, the videos indicated rat-typical “conscious” behaviours. However, 94 % (80/85) of the electrode-implantation -related seizures occurred in clusters, which in some cases compromised the electrographic assessment of sleep-wake state at the seizure onset.

The late seizures recorded during the 7th post-TBI month were unprovoked and did not include any additional factors like injury or anaesthesia (Fig. 1B,C – right panel). However, 71 % (67/95) of the seizures occurred in clusters, showing pathological interictal EEG, which compromised the analysis of sleep/wake stage in the EEG and analysis of conscious behaviours.

As the isoflurane anaesthesia that was used during injury induction and electrode operation can affect the occurrence, duration, and behavioural features of seizures, its duration was recorded. In the EEG cohort, the average anaesthesia duration on D0 during injury induction followed by electrode implantation was 110 ± 31 min (median 110 min, range 72–147 min). In the MRI cohort, anaesthesia duration related to electrode implantation during the 6th post-injury month was 79 ± 29 min (median 80 min, range 35–120 min).

### 3.2. Feature library of behavioural seizures

To extract the spectrum of behavioural features related to electrographic seizures, we first viewed all seizures included in the study. The consequent feature library included 59 different behavioural features **(**Table 1 and Table S1, Videos 1–10 in Supplementary material). The features were categorised into those occurring before the onset (5 s epoch; 3 features), during the ictal phase (43 features), or the postictal phase (30 s epoch, 13 features).

#### Pre-ictal phase.

Three different behavioural states were found during the preceding 5 s of electrographic seizure onset: awake, sleep, and immobility. In case of immobility, sleep/wake stage could not be defined in 77 % (114/149) of early, 20 % (17/85) of post-implantation, and 39 % (37/95) of late seizures due to a pathological EEG.

#### Ictal phase.

Altogether, 42 of 43 behavioural features listed in the feature library were found to be expressed during the ictal phase. No right (contralateral to injury) ear clonus was found, but the feature was included in the feature library for the sake of completeness. The features were categorized into the ones related to presumed consciousness level (7 features), to occurrence of movements in different body parts [mouth and whiskers (5), eyes (2), head (7), ears (2), paws (6), body and tail (12)], to autonomic features (2) or occurrence of wet-dog shakes (WDS). Up to 59 % (88/149) of early seizures showed no ictal behaviour other than immobility with or without arousal. Proportion of such post-implantation seizures was only 19 % (16/85) (59 % vs. 19 %, p < 0.001) and late seizures 8 % (9/95) (59 % vs. 9 %, p < 0.001). No difference was found between the post-implantation and late seizures (19 % vs. 9 %, p *>* 0.05).

**Fig. 2.**
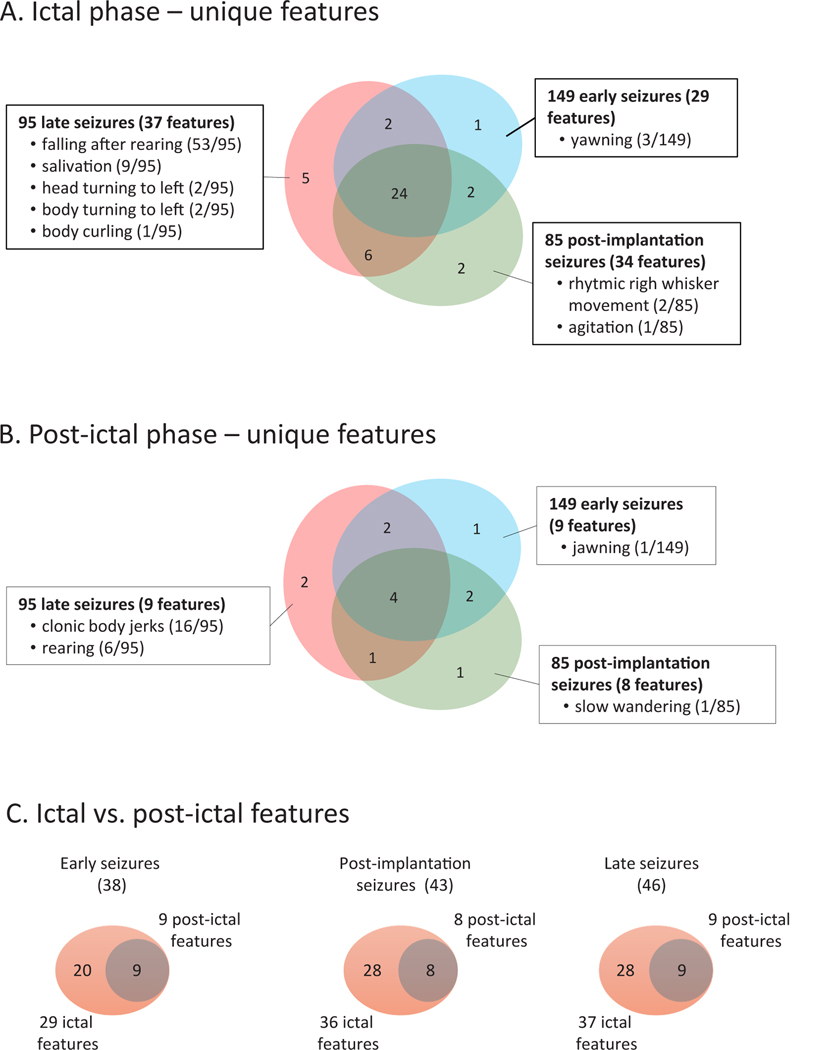
Ictal and/or post-ictal behavioral features of early, post-implantation, and late seizures have some unique behavioral features. **(A) *Ictal phase***. Of the 42 ictal features detected, 24 were found in both early, post-implantation, and late seizures. Twenty-six of the ictal features were found in both early and late seizures. Interestingly, 30 of the ictal features were common to post-implantation and late seizures. Only 1 feature (yawning) was unique to early seizures and 2 features to post-implantation seizures. Late seizures had 5 unique features, of which “falling after rearing” was the most common, and found in 56 % (53/95) of the late seizures. When early and late seizures were compared, 3 ictal features were unique to early seizures and 11 to late seizures, suggesting enrichment of seizure phenotype during epileptogenesis (see the list of features in each seizure category in Table 2). Post-implantation seizures had only four unique features compared to late seizures, making them difficult to behaviorally differentiate from the diagnostic late seizures. **(B) *Post-ictal phase (30 s after the end of the electrographic seizure) of early, post-implantation, and late seizures***. All seizure categories showed 8–9 post-ictal features, of which 4 were common. The unique post-ictal features were rare for early and post-implantation seizures. However, 17 % (16/95) of the late seizures showed clonic body jerks as a post-ictal feature not found in other seizure categories. **(C) *Ictal to post-ictal transition***. Ictal (the last 5 s of the ictal phase) vs. post-ictal (the first 5 s after electrographic seizure discontinuation) behaviors of early, post-implantation, and late seizures. Note the remarkable overlap in feature types, indicating that the electrographic end of the seizure could not be reliably determined by behavioral monitoring.

Further analysis revealed that 56 % (24/43) of the different behavioural features expressed during the ictal phase were found in both early, post-implantation, and late seizures (Fig. 2A). Of the features expressed during early and post-implantation seizures, 70 % (26/37) were common. Of the features expressed during early and late seizures, 65 % (26/40) were common. Of the features expressed during post-implantation and late seizures, 73 % (30/41) were common. Consequently, 1 feature was unique for early seizures (yawning), 3 for post-implantation seizures (rhythmic right whisker movement, freezing, agitation), and 5 for late seizures (falling and rearing, salivation, head turning to the left, body turning to the left, body curling). Typically, the unique features in early and post-implantation seizure categories were rare, being present in < 5 % of the seizures (Table 2). The exceptions were falling after rearing that associated with 56 % (53/95) and salivation that associated with 9 % (9/95) of the late seizures (Fig. 2A, Table 2).

#### Post-ictal phase.

The analysis of the post-ictal phase included 30 s immediately following the end of an electrographic seizure. Animals expressed 13 different behaviours during the post-ictal phase, all of which were also found during the ictal phase. Typically, the unique post-ictal features were rare, being present in < 6 % of the seizures. The exception was clonic body jerks, which were associated with 9 % (16/95) of late seizures (Fig. 2B, Table 2).

### 3.3. Behavioural characteristics of early, post-implantation, and late seizures

**Fig. 3.**
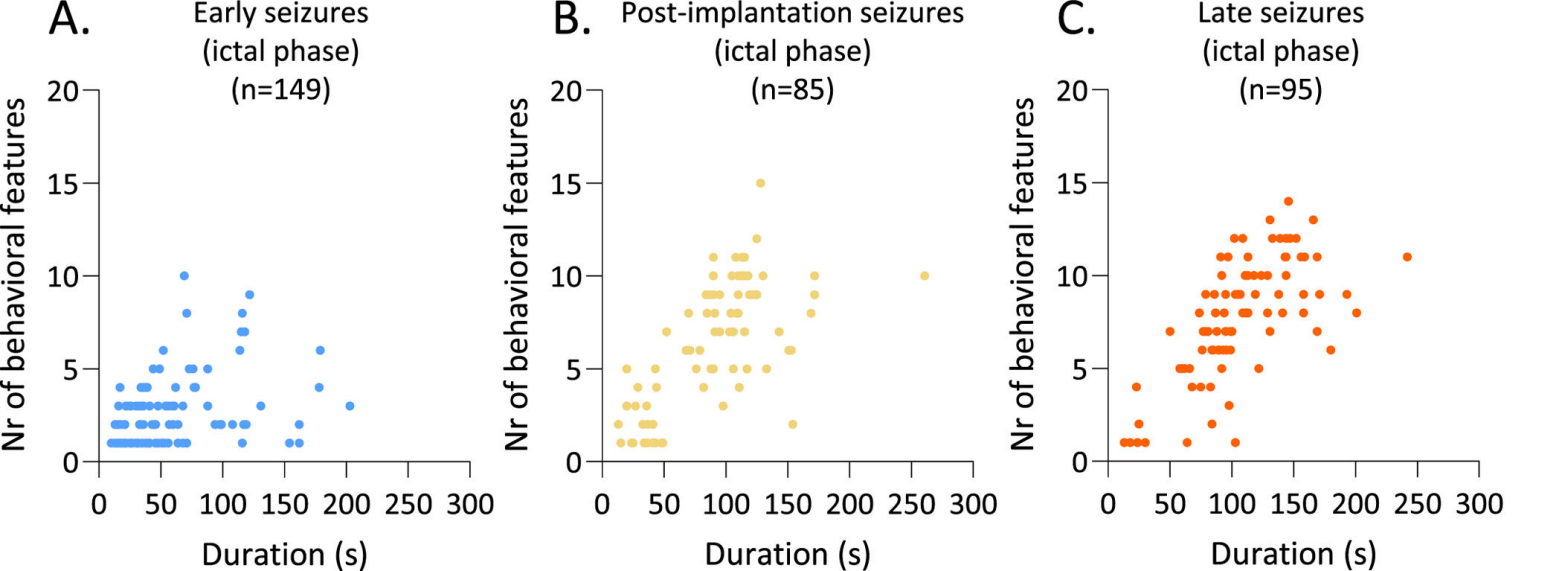
Duration of seizures vs. number of behavioral features during ictal phase. When all seizures were combined, the longer the seizure duration, the greater the average number of behavioural features [all seizures: n = 329, rho 0.624, p < 0.001]. Consequently, we analysed each seizure category separately. **(A)** Early seizures (n = 149). The average duration of the ictal phase (x-axis) was 47 ± 39 s, during which the average number of behavioral features (y-axis) was 2.2 ± 1.8. The longer the duration of seizures, the greater the number of behavioral features (rho 0.515, p < 0.001). **(B)** Post-implantation seizures (n = 85). The average duration of the ictal phase was 88 ± 46 s (p < 0.001 compared to early seizures), during which the average number of behavioral features was 6.2 ± 3.6 (p < 0.001 compared to early seizures). The longer the duration of seizures, the greater the number of behavioral features (rho 0.705, p < 0.001). **(C)** Late seizures (n = 95). The average duration of the ictal phase was 106 ± 42 s (p < 0.001 compared to early and p *>* 0.05 compared to post-implantation seizures), during which the average number of behavioral features was 7.6 ± 3.2 (p < 0.001 compared to early seizures; p = 0.054 compared to post-implantation seizures). The longer the duration of seizures, the greater the number of behavioral features (rho 0.705, p < 0.001).

**Fig. 4.**
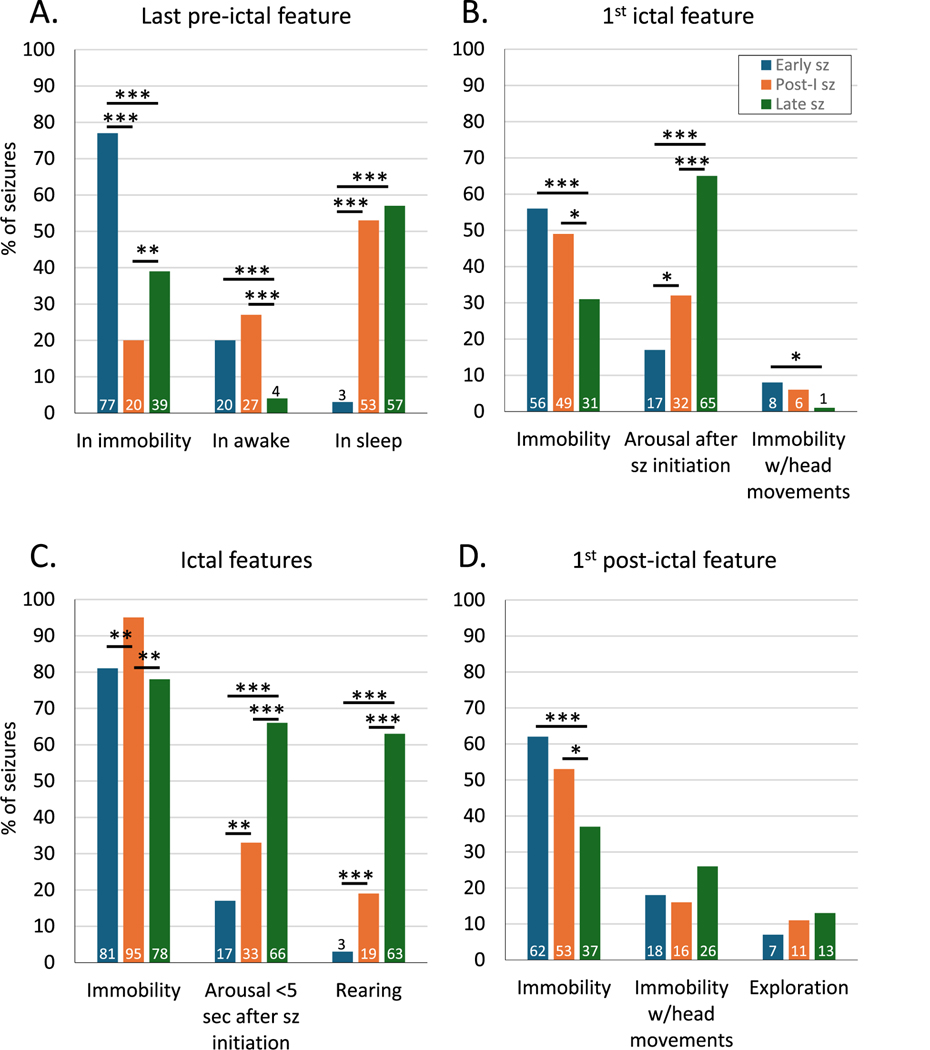
Comparison of the occurrence of the most common pre-ictal, ictal, and post-ictal features in different seizure categories (early, post-implantation, and late seizures). **(A)** The seizure onset occurred either during immobility (sleep-wake state could not be defined), awake, or sleep (numbers inside the bar refer to the occurrence in percentages). When the seizure onsets were compared within the seizure category, early seizures started most often in immobility than in awake (77 % vs. 20 %, p < 0.001) or in sleep (77 % vs. 3 %, p < 0.001). In cases in which the sleep stage could be defined, seizure initiation in awake was more common than in sleep (20 % vs. 3 %, p < 0.001). Unlike early seizures, post-implantation seizures started more often in sleep than in immobility (53 % vs. 20 %, p < 0.001) or in awake (53 % vs. 27 %, p < 0.01). Similar to post-implantation seizures, late seizures also started more often in sleep than in immobility (57 % vs. 39 %, p < 0.05) or in awake (57 % vs. 4 %, p < 0.001). When the three seizure categories were compared with each other, seizure initiation in sleep was typical of post-implantation and late seizures as compared to that of early seizures (see statistics in Panel A). (B) In all three seizure categories, the three most common behavioral features after electrographic seizure onset included immobility, arousal after (< 5 s) seizure initiation, and immobility with head movements. When the occurrences of the 1st ictal features were compared within the seizure category, early seizures typically started more often with immobility as compared to arousal after (< 5 s) seizure initiation (56 % vs. 17 %, p < 0.001) or immobility w/head movements (56 % vs. 8 %, p < 0.001). Post-implantation seizures also started more often with immobility than arousal after (< 5 s) seizure initiation (49 % vs. 32 %, p < 0.05) or immobility w/head movements (49 % vs. 6 %, p < 0.001). Similar to late seizures, arousal after (< 5 s) seizure initiation was more common than immobility w/head movements (32 % vs. 6 %, p < 0.001). Late seizures started more often with arousal after (< 5 s) seizure initiation than immobility (65 % vs. 31 %, p < 0.001) or immobility w/head movements (65 % vs. 1 %, p < 0.001). When the three seizure categories were compared with each other, arousal after (< 5 s) seizure initiation was a typical 1st ictal feature in late seizures as compared to that of early or post-implantation seizures (see statistics in Panel B). (C) The three most common ictal features occurring during late seizures were immobility, arousal after (< 5 s) seizure initiation, and rearing (no difference in occurrence). When the occurrences of the ictal features were compared within the seizure category, in early seizures, immobility occurred more often during the seizure than arousal after seizure initiation (81 % vs. 17 %, p < 0.001) or rearing (81 % vs. 3 %, p < 0.001). Also, in post-implantation seizures, immobility was a more common ictal feature than arousal after seizure initiation (95 % vs. 33 %, p < 0.001) or rearing (95 % vs. 19 %, p < 0.001). However, in late seizures, all three features were equally common (p *>* 0.05). When the three seizure categories were compared with each other, both arousal after (< 5 s) seizure initiation and rearing were more common in late than in early or post-implantation seizures (see statistics in Panel C). (D) The three most common first post-ictal features occurring during late seizures were immobility, immobility with head movements, and exploration. When the occurrences of the 1st post-ictal features were compared within the seizure category, in early seizures, immobility was more common than immobility w/head movements (62 % vs. 18 %, p < 0.001) or exploration (62 % vs. 7 %, p < 0.001). Similar to early seizures, in post-implantation seizures, immobility was a more common fir^st^ post-ictal feature than immobility w/head movements (53 % vs. 16 %, p < 0.001) or exploration (53 % vs. 11 %, p < 0.001). Also, in late seizures, immobility was a more common first post-ictal feature than exploration (37 % vs. 13 %, p < 0.001). When the three seizure categories were compared, immobility was more common 1st post-ictal feature in early than in post-implantation or late seizures (see statistics in Panel D). Statistical significances: ***, p < 0.001; **, p < 0.01; *, p < 0.05 (between seizure categories, χ2-test). Abbreviations: sz, seizure; WDS, wet-dog-shake.

**Fig. 5.**
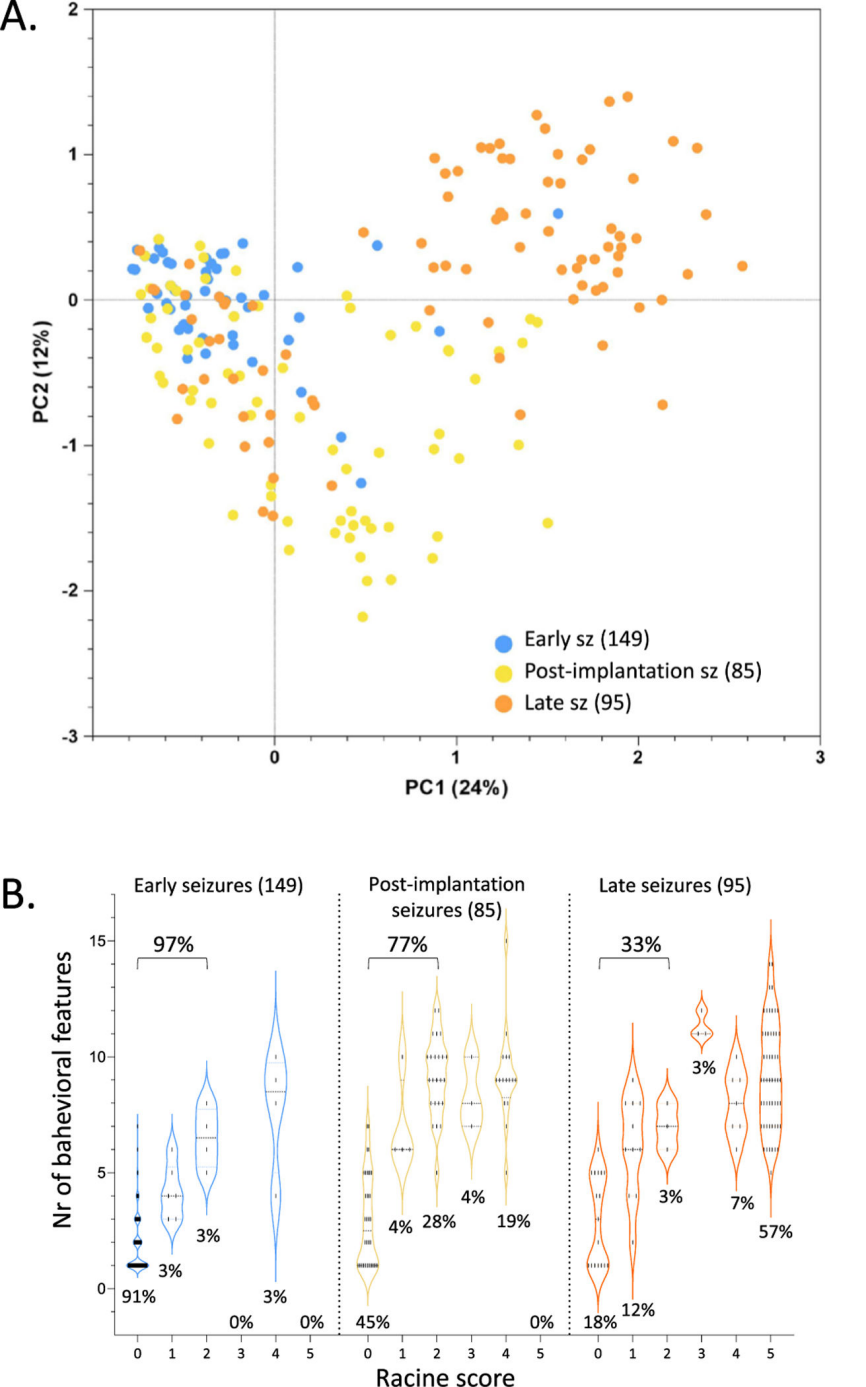
PCA of behavioral features and Racine scale in different seizure categories. **(A)** Principal component analysis revealed separation of behavioral phenotypes between early, post-implantation, and late seizures. Each dot refers to one seizure, with the location in the graph determined by the first two principal components (PC1 and PC2). Note that the 149 early seizures are clustered behind each other in a 2D graph, preventing us from seeing all the dots. PC1 (x-axis) accounts for the largest variance (24 %) and was driven by ictal behaviors with a motor component, including rearing (loading 0.504), piano playing (0.342), and arousal after seizure initiation (0.328). The behaviors contributing the most to PC2 (y-axis, 12 %) include wet-dog shakes (−0.292), left forepaw clonus (−0.220), and tonic head extension (−0.199). Interestingly, the early seizures (blue, n = 149) cluster towards the negative end of PC1, indicating less complex motor behaviors. Post-implantation seizures (yellow, n = 85) cluster more towards early seizures. Late seizures (orange, n = 95) cluster towards the positive end of PC1, indicating a higher occurrence of motor behaviors. All group comparisons were significant on PC1, with large inter-group distances (one-way ANOVA for PC1: F(2,326) = 132.4, adjusted p < 0.001), particularly between early and late seizures (adjusted p < 0.001). Also, there was a difference between the early and post-implantation seizures (adjusted p < 0.001) and between the post-implantation and late seizures (adjusted p < 0.001). Group comparisons on PC2 revealed group differences (F2, 326) = 55.6, adjusted p < 0.001). Group analysis indicated differences between the early and post-implantation seizures (adjusted p < 0.001) and between post-implantation and late seizures (adjusted p < 0.001). **(B) Left panel - *Racine score of early seizures (n*** = ***149)***. Of the 149 seizures, 91 % (135/149) had a Racine score of 0, even though rats showed up to 7 ictal behaviors. Note that none of the early seizures had a Racine score of 3 or 5. The greater the Racine score, the greater the number of behavioral features expressed (rho 0.496, p < 0.001). **(B) Middle panel - *Racine score of post-implantation seizures (n*** = ***85)***. Of the 85 seizures, 45 % (38/85) had a Racine score of 0, even though rats showed up to 7 ictal behaviors (45 % vs. 91 %, p < 0.001 compared to early seizures). Note that none of the post-implantation seizures had a Racine score of 5. The greater the Racine score, the greater the number of behavioral features expressed (rho 0.789, p < 0.001). **(C) Right panel - *Racine score of late seizures (n*** = ***95).*** Of the 95 seizures, only 18 % (17/95) had a Racine score 0, even though rats showed up to 6 seizure-related behaviors (18 % vs. 91 %, p < 0.001 compared to early seizures; 18 % vs. 45 %, p < 0.001 compared to post-implantation seizures). The majority of the late seizures were assigned a Racine score of 5. Altogether, 67 % of the late seizures were considered secondarily generalized (Racine score 3–5). The greater the Racine score, the greater the number of behavioral features expressed (rho 0.379, p < 0.001). Note that 97 % of the early and 77 % of the post-implantation seizures had Racine scores of 0–2, indicating they were focal. Only 33 % of late seizures had Racine scores of 0–2, which was less than that of early seizures (33 % vs. 97 %, p < 0.001) or post-implantation seizures (33 % vs. 77 %, p < 0.001), indicating that they were generalized.

We will first describe the type and frequency of behavioural characteristics during seizure onset, pre-ictal to ictal transition, ictal phase, ictal to post-ictal transition, and post-ictal phase of early, post-implantation, and late seizures. Data are summarised in Table 2, Tables S2–S5 and Figs. 2 and 3. Comparison in the occurrence of different features between the early, post-implantation and late seizures is described in Section 3.4.1–3.4.2, and data are summarised in Figs. 4 and 5.

#### 3.3.1. Early seizures (D0–D7 after TBI)

The 149 early seizures analysed occurred in 9 TBI rats with a total of 658 annotated behavioural features. The average number of features per early seizure was 4.4 ± 1.9 (median 4, range 3–13), including pre-ictal, ictal and post-ictal phases.

##### Pre-ictal phase.

Of the 149 early seizures, 77 % (114/149) started in “immobility”, which related to pathological EEG at the early post-injury phase, which did not allow for a reliable assessment of sleep-wake state (Table 2, Table S2**)**. Twenty-% (30/149) of seizures started in the awake state. Only 3 % (5/149) of early seizures started in EEG-confirmed sleep.

##### Transition from pre-ictal to ictal phase.

Data are summarised in Table S2. In cases with seizure onset in immobility (77 %, 114/149), the most common pre-ictal to ictal pair of behaviour was from immobility to immobility (67 %, 76/114), followed by from immobility to arousal (wake-up) within 5 s (18 %, 20/114). If the animal was awake at seizure onset (20 %, 30/149), the most common pre-ictal to ictal pairs of behaviour were from awake to immobility with head movements (30 %, 9/30) and from awake to immobility (27 %, 8/30). In cases with seizure onset in sleep (3 %, 5/149), the most common pre-ictal to ictal pair of behaviours was from sleep to arousal (wake-up) within 5 s (80 %, 4/5).

##### Ictal phase.

The average duration of the ictal phase was 47 ± 39 s (median 33 s, range 10–203 s). Altogether 313 behavioural features were annotated (Table 2). The average number of features per ictal phase was 2.1 ± 1.6 (median 2, range 1–9).

The 5 most common ictal behaviours after initiation of early electrographic seizure were immobility (56 %, 84/149), arousal (wake-up) within 5 s after seizure onset (17 %, 25/149), immobility with head movements (8 %, 12/149), unspecified body movement (5 %, 8/149) and arousal (wake-up) later (*>* 5 s) after initiation of electrographic seizure (5 %, 8/149) (Table S3). Overall, immobility occurred in 81 % (121/149) of seizures, immobility with head movements in 25 % (37/149), arousal within 5 s after seizure initiation in 17 % (25/149), and unspecified body movement in 11 % (17/149) of the early seizures (Table 2). The longer the early seizure duration, the greater the number of behavioural features (n = 149, rho 0.516, p < 0.001 (Fig. 3A).

##### Transition from ictal to post-ictal phase.

The most common behavioural features at the end of the ictal phase were immobility (65 %, 97/149), immobility with head movements (13 %, 20/149), and exploration with sniffing (7 %, 10/149) (Table S4). The most common behaviours at the beginning of the post-ictal phase were immobility (62 %, 93/149), immobility with head movements (18 %, 27/149), exploration (7 %, 10/149), and exploration with sniffing (7 %, 10/149) (Table S5).

In 83 % (123/149) of the electrographic seizures, there was no behavioural change from ictal to post-ictal phase (Table S4). If there was a behavioural change, the most common ictal to post-ictal pair of behaviours was from immobility to immobility (58 %, 87/149), followed by from immobility with head movements to immobility with head movements (12 %, 18/149) and exploration with sniffing to exploration with sniffing (7 %, 10/149) (Table S4). In fact, all (100 %, 9/9) different behavioural features expressed at the end of the ictal phase were also found in the post-ictal phase (Fig. 2C).

##### Post-ictal phase.

During the 30-s post-ictal phases of 149 early seizures, altogether 196 behavioural features were annotated (Table 2). The average number of features per post-ictal phase was 1.3 ± 0.5 (median 1, range 1–4). Further, 31 % (4/13) of the different behavioural features expressed during the post-ictal phase of early seizures were also found in post-implantation and late seizures (Fig. 2B). Seventy-five % (6/8) of the post-ictal features expressed after early seizures were common with those of the post-implantation, and 67 % (6/9) with those of the late seizures. Consequently, only 1 post-ictal feature was unique for early seizures (yawning) (Fig. 2B).

#### 3.3.2. Post-implantation seizures (D0–D7 after electrode implantation surgery in the 6th month)

The 85 post-implantation seizures analysed occurred in 10 TBI rats with a total of 734 annotated features (Table 2). The average number of features per post-implantation seizure was 8.6 ± 3.8 (median 9, range 3–16), including pre-ictal, ictal and post-ictal phases.

##### Pre-ictal phase.

Of the 85 post-implantation seizures, 53 % (45/85) started in sleep and 27 % (23/85) in the awake state (p < 0.001) (Table 2, Table S2). Only 20 % (17/85) were categorised to start in immobility, which was typically found in rats with seizure clusters.

##### Transition from pre-ictal to ictal phase.

Data are summarised in Table S2. In cases with seizure onset in sleep (53 %, 45/85), the most common pre-ictal to ictal pair of behaviours was from sleep to arousal (wake-up) within 5 s (51 %, 23/45), followed by from sleep to immobility (44 %, 20/45). If the animal was awake at seizure onset (27 %, 23/85), the most common pre-ictal to ictal pairs of behaviours were from awake to immobility (48 %, 11/23) and from awake to immobility with head movements (22 %, 5/23). In cases with seizure onset in immobility (20 %, 17/85), the most common pre-ictal to ictal pair of behaviours was from immobility to immobility (65 %, 11/17), followed by from immobility to arousal (wake-up) within 5 s (24 %, 4/17).

##### Ictal phase.

The average duration of the ictal phase was 88 ± 46 s (median 91 s, range 13–261 s), During the 85 ictal phases, 499 behavioural features were annotated (Table 2). The average number of features per ictal phase was 5.9 + 3.4 (median 6, range 1–14).

The most common first ictal behaviour after initiation of electrographic post-implantation seizure was immobility (49 %, 42/85), followed by arousal (wake-up) within 5 s (32 %, 27/85), and immobility with head movement (6 %, 5/85) (Table S3). Overall, the most common features during the course of the post-implantation seizures were immobility that occurred in 95 % (81/85) of seizures, WDSs (48 %, 41/85), exploration with sniffing (40 %, 34/85), and left (ipsilateral) forepaw clonus (40 %, 34/85) (Table 2). The longer the post-implantation seizure duration, the greater the number of behavioural features (n = 85, rho 0.684, p < 0.001) (Fig. 3B).

##### Transition from ictal to post-ictal phase.

The most common behavioural features at the end of the ictal phase were immobility (49 %, 42/85), wet-dog shakes (22 %, 19/85), and immobility with head movements (8 %, 7/85) (Table S4). The most common behaviours at the beginning of the post-ictal phase were immobility (53 %, 45/85), immobility with head movements (16 %, 14/85), wet-dog shakes (15 %, 13/85), and exploration (11 %, 9/85) (Table S5).

In 64 % (54/85) of the electrographic seizures, there was no behavioural change from the ictal to post-ictal phase (Table S4). If there was a behavioural change, the most common ictal to post-ictal pair of behaviours was from immobility to immobility (76 %, 32/42), followed by from wet-dog shakes during seizure to wet-dog shakes after seizure (53 %, 10/19), and from immobility with head movements to immobility with head movements (71 %, 5/7) (Table S4). Further analysis revealed that all (100 %, 8/8) different behavioural features expressed at the end of the ictal phase were also found in the post-ictal phase (Fig. 2C).

##### Post-ictal phase.

During the 85 post-ictal phases, 150 behavioural features were annotated (Table 2). The average number of features per post-ictal phase was 1.8 ± 0.9 (median 2, range 1–4). Of the post-ictal features expressed after post-implantation seizures 63 % (5/8) were common with late seizures (Fig. 2B). Only 1 post-ictal feature was unique for post-implantation seizures (slow wandering) (Fig. 2B).

#### 3.3.3. Late seizures

The 95 late seizures analysed occurred in 15 TBI rats with a total of 948 annotated features. The average number of features per seizure was 10 ± 3.3 (median 10, range 3–17), including pre-ictal, ictal and post-ictal phases.

##### Pre-ictal phase.

Like the post-implantation seizures, a majority of late seizures (57 %, 54/95) started during sleep (57 % vs. 53 %, p *>* 0.05) (Table 2, Table S2). However, 39 % (37/95) of the late seizures were categorised as starting in immobility, as it was not possible to define the sleep-wake state in the interictal EEG, which was typical of cases with seizure clusters. Only 4 % (4/95) of late seizures started during awake (Table 2**)**.

##### Transition from pre-ictal to ictal phase.

In 95 late seizures, the most common ictal behaviours after initiation of electrographic seizure were arousal (wake-up) within 5 s after seizure initiation (65 %, 62/95) and immobility (31 %, 29/95) (Table S2). In cases with seizure onset in sleep (57 %, 54/95), the most common pre-ictal to ictal pair of behaviours was from sleep to arousal (wake-up) within 5 s (70 %, 38/54), followed by from sleep to immobility (30 %, 16/54) (Table S2). In cases with seizure onset in immobility (39 %, 37/95), the most common pre-ictal to ictal pairs of behaviours were from immobility to arousal (wake-up) within 5 s (65 %, 24/37) and immobility to immobility (32 %, 12/37) (Table S2). If the animal was awake at seizure onset (4 %, 4/95), the most common pre-ictal to ictal pairs of behaviours were from awake to immobility with head movements, to immobility, to exploration, and to exploration with sniffing (all 25 %, 1/4) (Table S2).

##### Ictal phase.

The average duration of the ictal phase was 106 ± 42 s (median 99 s, range 13–242 s). During the 95 ictal phases, 684 behavioural features were annotated (Table 2). The average number of features per ictal phase was 7.2 + 3.1 (median 7, range 1–14).

The most common first ictal behaviours after initiation of electrographic post-implantation seizure were arousal after (< 5 s) seizure initiation (65 %, 62/95) and immobility (31 %, 29/95) (Table S3). Overall, the most common ictal features during the seizure were immobility (78 %, 74/95), arousal after (< 5 s) seizure initiation (66 %, 63/95), rearing (64 %, 61/95), and piano playing (55 %, 52/95) (Table 2). The longer the late seizure duration, the greater the number of behavioural features (n = 95, rho 0.624, p < 0.001) (Fig. 3C).

##### Transition from ictal to post-ictal phase.

The most common behavioural features at the end of the ictal phase were immobility (41 %, 39/95), rearing (19 %, 18/95), and immobility with head movements (14 %, 13/95) (Table S4). The most common behaviours at the beginning of the post-ictal phase were immobility (37 %, 35/95), immobility with head movements (26 %, 25/95), exploration (13 %, 12/95), and wet-dog shakes (9 %, 9/95) (Table S5).

In 57 % (54/95) of the seizures, there was no behavioural change from the ictal to post-ictal phase. If there was a behavioural change, the most common ictal to post-ictal pair of behaviours was from immobility to immobility (62 %, 24/39), followed by from rearing to rearing (33 %, 6/18) and from immobility with head movements to immobility with head movements (92 %, 12/13) (Table S4). Further analysis revealed that all (100 %, 9/9) different behavioural features expressed at the end of the ictal phase were also found in the post-ictal phase (Fig. 2C).

##### Post-ictal phase.

During the 95 post-ictal phases, 169 behavioural features were annotated (Table 2). The average number of features per post-ictal phase was 1.8 ± 0.7 (median 2, range 1–4). Two of the post-ictal features were unique for late seizures (clonic body jerks, rearing) (Fig. 2B).

### 3.4. Differentiation of early, post-implantation and late seizures based on seizure phenotype

#### 3.4.1. Duration of ictal phase and feature numbers

##### Seizure duration.

The average duration of early seizures was shorter than that of post-implantation seizures (47 ± 39 s *vs.* 88 ± 46 s, p < 0.001) or late seizures (47 ± 39 s *vs.* 106 ± 42 s, p < 0.001). Duration of post-implantation and late seizures did not differ (88 ± 46 s vs. 106 ± 42 s, p *>* 0.05).

##### Ictal feature number.

The average number of features expressed during ictal phase of post-implantation and late seizures was comparable [5.9 ± 3.4 vs. 7.2 ± 3.1, p *>* 0.05]. However, both the post-implantation and late seizures had more features than the early seizures [5.9 ± 3.4 vs. 2.1 ± 1.6, 7.2 ± 3.1 vs. 2.1 ± 1.6, respectively, both p < 0.05].

##### Post-ictal feature number.

The number of post-ictal features expressed during 30 s after the end of electrographic early seizures was comparable to that of post-implantation and late seizures (1.3 ± 0.5 vs. 1.8 ± 0.9 vs. 1.8 ± 0.7, respectively, all comparisons p *>* 0.05).

#### 3.4.2. Prevalence of pre-ictal, ictal and post-ictal features

Comparison of the feature occurrence within and between the seizure categories is summarised in Fig. 4.

##### Pre-ictal.

The seizure onset occurred either during immobility (sleep-wake state could not be defined), awake, or sleep (Fig. 4A). Typically, early seizures occurring during the first post-injury week started in immobility, which was more common than in post-implantation (77 % vs. 20 %, p < 0.001) or late (77 % vs. 39 %, p < 0.001) seizures. Only 3 % of early seizures started in EEG-confirmed sleep, which was substantially less than that in post-implantation (3 % *vs.* 53 %, p < 0.001) or late seizures (3 % *vs.* 57 %, p < 0.001). Unlike early seizures, the post-implantation and late seizures occurring months after TBI started in sleep (53 % post-implantation vs. 3 % early, p < 0.001; 57 % late vs. 3 % early, p < 0.001). Notably, only 4 % of late seizures started during awake, which was less than that in early (4 % vs. 20 %, p < 0.001) or post-implantation (4 % vs. 27 %, p < 0.001) seizures.

##### Seizure onset.

In all three seizure categories, the three most common first behavioural features after electrographic seizure onset were immobility, arousal after (< 5 s) seizure initiation, and immobility with head movements (Fig. 4B). However, immobility was the more common early ictal feature in early and post-implantation (no difference, p *>* 0.05) than late seizures (56 % early vs. 31 % late, p < 0.001; 49 % post-implantation vs. 31 % late, p < 0.05). Arousal after seizure initiation was a more common early ictal feature in late than in other seizure categories (65 % late vs. 17 % early, p < 0.001; 65 % late vs. 32 % post-implantation, p < 0.001).

##### Ictal features during seizure progression.

The three most common ictal features occurring during the course of late seizures were immobility, arousal after (< 5 s) seizure initiation, and rearing (Fig. 4C). Of these, immobility was frequent in all seizure categories. However, features that were common in late seizures but less often observed in post-implantation or early seizures included arousal after (< 5 s) seizure initiation (66 % late vs. 33 % post-implantation, p < 001; 66 % late vs. 17 % early, p < 0.001), and rearing (63 % late vs. 19 % post-implantation, p < 0.001; 63 % late vs. 3 % early, p < 0.001).

##### Post-ictal.

The three most common first post-ictal features occurring after the end of the electrographic late seizure were immobility, immobility with head movements, and exploration (Fig. 4D). Immobility was the most common first post-ictal feature across all seizure types, even though less common in late than in early (37 % late vs. 62 % early p < 0.001) or post-implantation (37 % late vs. 53 % post-implantation, p < 0.05) seizures.

#### 3.4.3. Principal component analysis

Data are summarised in Fig. 5A. To expand the analysis from individual behavioural features to the overall pattern of behavioural seizure phenotype, we performed the Principal Component Analysis (PCA). The PCA revealed separation of behavioural phenotypes between early, post-implantation, and late seizures. The PCA1 (24 % of the variance) was driven by ictal behaviours with a motor component, including rearing (loading 0.504), piano playing (0.342), and arousal after seizure initiation (0.328). The behaviours contributing the most to PC2 (12 %) included wet-dog shakes (−0.292), left forepaw clonus (−0.220), and tonic head extension (−0.199). The early seizures clustered towards the negative end of PC1 with less complex motor behaviours. Post-implantation seizures clustered more towards early seizures. Late seizures clustered towards the positive end of PC1, indicating a higher occurrence of motor behaviours. All group comparisons were significant on PC1, with large inter-group distances (one-way ANOVA for PC1: F (2,326) = 132.4, adjusted p < 0.00), particularly between early and late seizures (adjusted p < 0.001). Also, there was a difference between the early and post-implantation seizures (adjusted p < 0.001) and between the post-implantation and late seizures (adjusted p < 0.001). Group comparisons on PC2 revealed group differences (F2, 326) = 55.6, adjusted p < 0.001), indicating differences between the early and post-implantation seizures (adjusted p < 0.001) and between post-implantation and late seizures (adjusted p < 0.001).

#### 3.4.4. Racine score

Data are summarised in Fig. 5B.

##### Early seizures.

The average Racine score of early seizures was 0.2 ± 0.7 (median 0, n = 149). The greater the Racine score, the greater the number of behavioural features (n = 149, r = 0.496, p < 0.001).

Most of the 149 early seizures (91 %, 135/149) had a Racine score of 0 (Fig. 5B). In seizures with Racine score 0 (n = 135), the average number of behavioural features annotated based on the feature library was 1.9 ± 1.2 (median 1, range 1–7). The main behavioural features included immobility (82 %, 110/135) and immobility with head movements (24 %, 32/135).

Only 9 % (14/149) of the seizures had a Racine score *>* 0. Of the 14 seizures, six were scored as Score 1, four as Score 2, and four as Score 4. The average number of features per seizure was 5.9 ± 2.2 (median 5.5, range 3–10). The main behavioural features included immobility (11/14, arousal (”wake-up”) after seizure initiation (10/14), head nodding (9/14), and Immobility with head movements (5/14).

##### Post-implantation seizures.

The average Racine score was 1.5 ± 1.5 (median 2, n = 85), which was greater than that of early seizures (1.5 ± 1.5 vs. 0.2 ± 0.7, p < 0.001) (Fig. 5B). The greater the Racine score, the greater the number of behavioural features (n = 85, rho 0.789, p < 0.001).

Almost half of the 85 post-implantation seizures (45 %, 38/85) had a Racine score of 0. The average number of behavioural features per seizure was 2.9 ± 1.9 (n = 38, median 2.5, range 1–7). The most common behavioural feature was immobility (92 %, 35/38).

Of the 85 post-implantation seizures, 55 % (47/85) had Racine score *>* 0, ranging from 1 to 4. Of these 47 seizures, four were scored as Score 1, 24 as Score 2, three as Score 3, and 16 as Score 4. The average number of features per seizure was 8.9 ± 1.9 (n = 47, median 9, range 5–15). The main behavioural features included immobility (43/47), left forepaw clonus (34/47), wet-dog shakes during seizure (32/47), and head nodding (31/47).

##### Late seizures.

The average Racine score was 3.4 ± 2.1 (n = 95, median 5, range 0–5), which was greater than that of early seizures (3.4 ± 2.1 vs. 0.2 ± 0.7, p < 0.001) or post-implantation seizures (3.4 ± 2.1 vs. 1.5 ± 1.5, p < 0.001) (Fig. 5B). The greater the Racine score, the greater the number of behavioural features (n = 95, rho 0.379, p < 0.001).

Only 18 % (17/95) of the late seizures had a Racine score of 0. The average number of behavioural features of the Score 0 seizure was 3.0 ± 1.9 (n = 17, median 3, range 1–6). The most common behavioural feature of the late seizures with Racine score 0 was immobility (100 %, 17/17).

A large majority of 95 late seizures (82 %, 78/95) had a Racine score *>* 0. Of the 78 seizures, 11 were categorised as Score 1, three as Score 2, three as Score 3, seven as Score 4, and 54 as Score 5. The average number of features per seizure was 8.7 ± 2.4 (n = 78, median 8, range 2–14). The main behavioural features included rearing (61/78), immobility (59/78), arousal (”wake-up”) after seizure initiation (58/78), falling after rearing (53/78), and piano playing (52/78).

Ninety-seven % of the early and 77 % of the post-implantation seizures had Racine scores of 0–2, indicating that a large majority of them were focal. Only 33 % of late seizures had Racine scores of 0–2, which was less than that of early seizures (33 % vs. 97, p < 0.001) or post-implantation seizures (33 % vs. 77 %, p < 0.001), indicating that most late seizures were generalised.

Taken together, both the PCA and Racine score analysis indicated behavioural differences between the early, post-implantation and late seizures, which need to be considered in seizure description and algorithm training.

## 4. Discussion

The present study was kindled by an observation that the commonly used Racine score covered only a fraction of behavioural features observed in seizures occurring after TBI. Therefore, we systematically reviewed the high-resolution vEEG recordings in our TBI cohorts to generate a library of behavioural features to more accurately describe behavioural characteristics (semiology) of electrographic seizures. The early, electrode-implantation -related and late seizures were assessed separately as they occurred at different post-TBI recovery time points. We had 4 major findings. First, 91 % of the early seizures, 45 % of the electrode-implantation -related seizures, and 18 % of the late seizures that had been scored as 0 using the Racine score showed up to 6–7 ictal-related behaviours when described using the newly developed behavioural feature library. Second, the behavioural features between the early and late seizures differed, suggesting evolution of seizure semiology throughout epileptogenesis, which needs to be considered in the optimisation of the training of automated seizure-detection algorithms. Third, seizures detected during the first week after electrode implantation in the chronic phase differed from the unprovoked late seizures, suggesting that electrode-implantation-related seizures could be considered provoked rather than diagnostic late seizures. Fourth, high-resolution video monitoring could not reliably differentiate transition from pre-ictal to ictal or ictal to post-ictal phases, emphasising the need for EEG monitoring in the definition of seizure onset, seizure end and seizure duration.

### 4.1. Richness of ictal behaviours in seizures after TBI

The proposed feature library for the description of ictal behaviours comprised 43 features. The feature library included all motor features that were included in the Racine scale (see Section 2; Racine, 1972). However, in our feature library, they were more itemised as the features and sequence of events in different body parts could be better defined in high-resolution videos. For example, the “paw clonus” was described in the limb where it occurred, and “rearing and falling” (stage 5) was divided into the components, if it appeared on video with additional features such as “piano playing” or “freezing in rearing position”. It should be mentioned that in the original description, Racine reported the occurrence of exploratory behaviour and immobility during after-discharges induced by amygdala stimulation (Racine, 1972). However, the non-motor features were not included in the Racine scale.

The need to add granularity to the feature list was also related to the observation that in rats with TBI, the spectrum of ictal behaviours appeared greater than that in chemoconvulsant or electrical stimulation-induced seizures (Veliskova and Velisek, 2017a, 2017b). For example, late seizures showed behaviours like hind limb clonus and body curling, which were not included in the Racine score. In particular, the Racine score was not applicable for the description of 91 % of early seizures, as they were largely non-convulsive and showed features such as arousal after seizure onset or slow horizontal scanning of the environment. Comparable to post-TBI early seizures, non-convulsive seizures were common after hippocampal electrical stimulation-induced status epilepticus in rats and mice (Avdic et al., 2018). Also, unprovoked early seizures after haemorrhagic stroke in rats showed comparable behavioural features to those after TBI, including disoriented walking with raised tail or with curved back and hind limb clonus (Germonpré et al., 2020). Tetanus-toxin-induced focal occipital late seizures in rats showed non-motor seizures with sudden freezing or agitated and aggressive searching behaviour (Chang et al., 2018). Recently, automatisms, autonomic features, and abnormal behaviours were reported to be common in cats with autoimmune encephalitis (Binks et al., 2025).

Taken together, our data proposed a need to expand the seizure semiology description to include features of non-convulsive seizures and even tailor the description according to the aetiology and location of the epileptogenic lesion. Further, assessment of post-injury behaviours using machine learning assisted video analysis could provide unforeseen opportunities to detect subtle behaviours to diagnose periods of non-convulsive seizures or status epilepticus after TBI as suggested by the study of Gschwind et al. (2023).

### 4.2. Detecting seizure onset without EEG

Like in humans, in animal models as well, the EEG is considered the gold standard for the detection of ictal onset (Bertram, 2017). However, implantation of epidural or intracerebral electrodes is not always possible, for example, if the experimental protocol includes magnetic resonance imaging (MRI) or other analyses that can be compromised by electrode material or the implantation procedure. Therefore, we tested the hypothesis that a change in behavioural features will reveal a transition from the pre-ictal to ictal phase. This idea was based on our initial screening of ictal behaviours, which indicated that in 65 % of late seizures that typically start in sleep, the animal woke up or showed arousal within 5 s after the onset of an electrographic seizure, agreeing with our previous observations (Andrade et al., 2019). In rare cases of an awake onset of late seizures, the ictal onset was followed by immobility with or without head movements. However, 27 % of the late seizures did not show any clear behavioural change during the pre-ictal to ictal transition. Consequently, the accurate time of seizure onset needed to be determined electrographically.

Similar to the late seizures during the 7th post-injury month, a majority (73 %) of seizures detected during the first week after electrode implantation, performed during the 6th post-injury month, started in sleep or immobility. However, arousal within 5 s after seizure initiation during the pre-ictal to ictal transition was observed in only 32 %, which was substantially less compared to 65 % in late seizures, suggesting differences between the brain networks involved in ictogenesis under the two conditions. Similar to late seizures, early ictal arousal was typical of seizures starting in sleep. In correlation with late seizures, about one-third (36 %) of the electrode-implantation-related seizures showed no clear behavioural change at ictal onset, challenging the assessment of seizure onset time by observation only.

In seizures detected during the 1st week post-injury, the transition from immobility to immobility was the most common feature during the pre-ictal to ictal transition. Only 17 % of the early seizures showed early ictal arousal. Like in late and post-implantation seizures, early ictal arousal was typical of early seizures that started in sleep. As a caveat to the analysis, most of the rats with early seizures or rats with electrode implantation or late seizures occurring in clusters (≥ 3 seizures per 24 h) had pathological EEG. Consequently, EEG was not useful for the analysis of sleep stages, and pre-ictal behavior was annotated as immobility. Also, correlating behavioral features of early seizures with EEG, which typically occurred during the first 48 h post-injury, was compromised by the limited ability of the animal to express various behaviors at the acute post-injury stage.

Taken together, while behavioral cues such as ictal arousal may assist in detecting the onset of late seizures, a large number of early, post-implantation, and late seizures lack a distinct behavioral transition that would enable the detection of seizure onset without EEG.

### 4.3. Detecting seizure termination without EEG

Human studies have shown variability in electrographic patterns of seizure termination, with a majority showing synchronous termination and the rest being asynchronous or, in rare cases, unclassified (Fisher et al., 2014; Salami et al., 2022). Behaviourally, seizure cessation is often characterised by disorienting symptoms such as confusion, drowsiness, hypertension, headache, or nausea (Abood and Bandyopadhyay, 2025). However, limitations related to accuracy in detecting the ictal to post-ictal transition based on observation only without EEG monitoring are well acknowledged (Kinney et al., 2019).

To our knowledge, no previous studies have addressed the behaviours related to the ictal to post-ictal transition in rodent models of structural epilepsies. In the present analysis, in 70 % of all seizures detected, there was no behavioural change associated with electrographically determined ictal to post-ictal transition. Particularly, 83 % of early, 64 % of post-implantation, and 57 % of late seizures lacked a behavioural change from ictal to post-ictal phase, the ictal immobility to post-ictal immobility being the primary transitional behaviour in all seizure categories.

These findings show that behavioural cues alone lack the precision needed to reliably indicate the end of the seizure.

### 4.4. Progression of seizure semiology along epileptogenesis

Analysis of behavioural characteristics between early and late seizures indicates that the late seizures were longer and had a greater number of behavioural features than the early seizures. Further, the late seizures showed several ictal features not observed in early seizures, separating the two seizure categories from each other. This suggests that throughout the epileptogenic process, networks involved in the generation of ictal activity expand, which is in correlation with previous follow-up imaging studies in electrogenesis (De Feo et al., 2022; Shultz et al., 2013a, 2013b).

In addition to late seizures justifying the diagnosis of PTE at the chronic phase, we observed seizures during the first 84 h after electrode implantation, which was performed about 6 months post-TBI. Comparison of post-implantation and late seizures recorded about 1 month apart in the chronic phase indicated that the average duration of seizures or the number of behavioural features during the ictal phase did not differ, and 73 % of the ictal features were common to post-implantation and late seizures. However, late and post-implantation seizures showed differences in the occurrence of pre-ictal, ictal, and post-ictal features. Moreover, late seizures showed 7 features not found in post-implantation seizures, typically indicating secondary generalisation, that is, falling after rearing, which was not observed in any of the post-implantation seizures. When compared to early seizures, the temporal occurrence of post-implantation seizures within 80 h post-surgery/anaesthesia matched that of early seizures. However, the complexity of the ictal behavioural features of post-implantation seizures was greater than that of early seizures, possibly related to brain recovery by the 6th post-injury month, allowing the generation of complex behavioural seizure phenotypes.

Detection of post-implantation seizures raised a question: are these seizures induced/provoked, or can they be considered diagnostic for PTE? The post-implantation seizures were recorded about 6 months after TBI, excluding the contribution of the acute TBI effect on seizure-induction. One etiologic factor for post-implantation seizures could be damage to the blood–brain barrier (BBB) caused by isoflurane anaesthesia that was used during implantation of epidural/intracerebral electrodes. Previous experimental studies have demonstrated that isoflurane causes a dose-dependent opening of the blood–brain barrier (BBB) that is initiated in the thalamus and at higher concentrations also involves cortical structures (Tétrault et al., 2008). The new BBB damage induced by intracerebral/epidural electrode implantation in isoflurane anesthetized rats on the 6th post-injury month, together with existing TBI-induced chronic BBB damage, could augment the leakage of plasma proteins to the extracellular space and lead to hyperexcitability and seizures (Friedman, 2011; Savotchenko et al., 2023; van Vliet et al., 2020). Interestingly, clinical studies have also shown that inhalation anaesthesia with isoflurane or sevoflurane can be ictogenic (Harrison, 1986; Hymes, 1985; Poulton and Ellingson, 1984). However, post-electrode implantation seizures can occur in sham-operated and even in naïve rats with epidural electrodes only (Andrade et al., 2019; Di Sapia et al., 2021; Fig. S1). Therefore, the contribution of electrode implantation-related trauma – even when being epidural - to ictogenesis remains to be further explored to decide whether seizures detected during the first week after electrode implantation should be considered “provoked” and not diagnostic for PTE.

Taken together, the evolution of the behavioural complexity of seizure semiology after TBI suggests expansion of the ictogenic network over time. The occurrence of seizures after procedures (e.g., electrode implantation) requiring isoflurane anaesthesia should be monitored and probably considered as induced seizures rather than unprovoked diagnostic late seizures.

### 4.5. Methodological limitations

To address the spectrum of various behavioural seizure types, we analysed TBI rats with acute seizures during the first post-injury week and late seizures during the 7th month. In addition, we assessed TBI rats with seizures during the first week after electrode implantation in the 6th post-injury month. Animal numbers in each seizure category varied from 9 to 15. This could raise a concern that animal-specific seizure-related behaviours could have introduced a bias into the analysis. Therefore, the completeness of the feature list needs to be monitored and complemented if new features are detected in PTE models or in other models with spontaneous seizures. Adding testing of consciousness and monitoring of autonomic functions can provide additional information to seizure analysis. We have recently shown that rats can vocalise at ultrasonic frequencies during induced seizures (Lara-Valderrábano et al., 2022). Possible presence of vocalisations during unprovoked seizures remains to be explored and added to the behavioural feature library. Finally, technical issues such as the position of the animal relative to the camera can affect feature detection.

In the present analysis, 4 bilateral epidural and 6 ipsilateral intracerebral electrodes were used in video-EEG recordings. Therefore, unlike in the clinic, where seizure semiology can be aligned with high-resolution electrographic recordings with up to 256 electrodes, we did not make any serious attempt to match the seizure semiology with the seizure onset site or with the evolution of electrographic spread of ictal activity in a given animal.

Assessment of sleep-wake stage in EEG was challenging in some cases. During D0-D7, when early seizures were recorded, most of the animals in the analysis cohort exhibited periods of non-convulsive status epilepticus. Consequently, the severity of pathology in the EEG varied, and the sleep-wake stage could not be defined for all early seizures. At a chronic stage, a large number of post-implantation and late seizures belonged to seizure clusters. Due to pathological interictal EEG, the definition of sleep-wake stage at the onset of some of these seizures was not possible, and the state was considered “immobility”.

## 5. Conclusion

Our analysis shows that the spectrum of seizure-related behavioural features in seizures after TBI expands greatly beyond those included in the Racine scale, which is commonly applied in the description of behavioural seizures and used as a reference for training of machine learning algorithms for seizure detection. Rather than attempting to generate a new scale for seizure semiology, we propose to use a seizure library to individualize the behavioral description of seizures and treatment effects. This will increase the granularity in the description of preclinical seizure semiology and expand the glossary of existing common data elements (CDEs) used for harmonisation of the description of behavioural seizure characteristics in preclinical research, aligning with the objectives of the International League Against Epilepsy/American Epilepsy Society working group for common data elements (CDEs) (Barker-Haliski et al., 2022). The feature library presented can help to anticipate the behavioural patterns that need to be included in the training of artificial intelligence-guided video-based preclinical seizure detection algorithms, which are urgently needed to speed up non-invasive, affordable epilepsy diagnosis and assessment of treatment effects. Finally, as a significant number of post-TBI seizures did not show any major motor symptoms, machine learning guided approaches focusing on overall animal behaviour rather than seizure-related behaviour only [e.g., Moseq (Gschwind et al., 2023), Key-point Moseq (Weinreb et al., 2024), DeepLabCut (Mathis et al., 2018)] may turn out to be useful for the diagnosis of non-convulsive seizures and status epilepticus in animals with TBI and to monitor the treatment effects.

## Supplementary Material

1

2 Video S1. A video clip is available online. Supplementary material related to this article can be found online at doi:10.1016/j.jneumeth.2025.110671.

3 Video S2. A video clip is available online. Supplementary material related to this article can be found online at doi:10.1016/j.jneumeth.2025.110671.

4 Video S3. A video clip is available online. Supplementary material related to this article can be found online at doi:10.1016/j.jneumeth.2025.110671.

5

6 Video S4. A video clip is available online. Supplementary material related to this article can be found online at doi:10.1016/j.jneumeth.2025.110671.

7 Video S5. A video clip is available online. Supplementary material related to this article can be found online at doi:10.1016/j.jneumeth.2025.110671.

8 Video S6. A video clip is available online. Supplementary material related to this article can be found online at doi:10.1016/j.jneumeth.2025.110671.

9 Video S7. A video clip is available online. Supplementary material related to this article can be found online at doi:10.1016/j.jneumeth.2025.110671.

10 Video S8. A video clip is available online. Supplementary material related to this article can be found online at doi:10.1016/j.jneumeth.2025.110671.

11 Video S9. A video clip is available online. Supplementary material related to this article can be found online at doi:10.1016/j.jneumeth.2025.110671.

12 Video S10. A video clip is available online. Supplementary material related to this article can be found online at doi:10.1016/j.jneumeth.2025.110671.

## Figures and Tables

**Table 1:** Feature library listing the behavioural features (semiology) of the pre-ictal, ictal, and post-ictal phases of early, post-electrode implantation, and late seizures. A total of 3 pre-ictal, 43 ictal, and 13 post-ictal behavioural features were annotated based on high-resolution videos that were time-locked with electroencephalogram (EEG). Pre-ictal phase was defined as the preceding 5 s of electrographic seizure onset. The postictal phase included a 30-second period immediately following the end of an electrographic seizure. Description of the features is presented in Table S1. Illustrative video-EEG examples are shown in Video 2.

Category	Feature

**Pre-ictal phase (3)**	
	In awake
	In immobility
	sleep
**Ictal phase (43)**	
Consciousness (7)	Arousal (”wake-up”) after seizure initiation^1^ Arousal (”wake-up”) later during seizure^2^
	Immobility
	Immobility w/head movements
	Exploration
	Exploration w/sniffing
	Slow wandering
Mouth and whiskers (5)	
	Chewing
	Sniffing
	Rhythmic right whisker movement
	Rhythmic left whisker movement
	Rhythmic bilateral whisker movements
Eyes (2)	
	Left eye blinking
	Right eye blinking
Head (7)	
	Head turning to left
	Head turning to right
	Slow ”scanning” horizontal head movement
	Head nodding
	Head clonus
	Tonic head extension
	Yawning
Ears (2)	
	Left ear clonus
	Right ear clonus
Paws (6)	
	Left forepaw clonus
	Left hind paw clonus
	Bilateral forepaw clonus
	Piano playing
	Right forepaw clonus
	Right hind paw clonus
Body and tail (12)	
	Freezing
	Unspecified body movement
	Rearing
	Freezing in rearing position
	Falling after rearing
	Agitation
	Tonic body extension
	Clonic body jerks
	Body turning left
	Body turning right
	Body curling
	Tail extension
Autonomic (2)	
	Fast breathing
	Salivation
WDS (1)	
	WDS
**Post-ictal phase (13)**	
	Immobility
	Immobility w/head movements
	Slow wandering
	Slow ”scanning” horizontal head movement
	Exploration
	Rearing
	Agitation
	Chewing
	Yawning
	Salivation
	WDS
	Clonic body jerks
	Unspecified body movement

***Abbreviations:*** WDS, wet-dog shake;

1 arousal after seizure initiation refers to “wake-up” < 5 s after electrographic seizure initiation;

2 arousal later during seizure refers to “wake-up” > 5 s after electrographic seizure initiation. Number of features is shown in parenthesis.

**Table 2:** Types and frequencies of behavioral features occurring during the pre-ictal, ictal phase, and post-ictal phase in different seizure categories (149 early seizures, 85 post-implantation seizures, 95 late seizures). The pre-ictal phase was annotated during the 5 s before the onset of the electrographic seizure, the ictal phase during the electrographic seizure, and the post-ictal period during the 30 s after the end of the electrographic seizure.

Early seizures (149)			Post-implantation seizures (85)			Late seizures (95)		

Pre-ictal phase			Pre-ictal phase			Pre-ictal phase		
Feature (3)	%	#Sz	Feature (3)	%	#Sz	Feature (3)	%	#Sz
In immobility	77 %	114	In sleep	53 %	45	In sleep	57 %	54
In awake	20 %	30	In awake	27 %	23	In immobility	39 %	37
In sleep	3 %	5	In immobility	20 %	17	In awake	4 %	4
Ictal phase			Ictal phase			Ictal phase		
Feature (29)	%	#Sz	Feature (35)	%	#Sz	Feature (37)	%	#Sz
Immobility	81 %	121	Immobility	95 %	81	Immobility	78 %	74
Immobility w/head movements Arousal (”wake-up”) after seizure initiation^1^	25 % 17 %	37 25	WDS Left forepaw clonus	48 % 40 %	41 34	Arousal (”wake-up”) after seizure initiation Rearing	66 % 64 %	63 61
Unspecified body movement Arousal (”wake-up”) later during seizure^2^	11 % 9 %	17 13	Exploration w/sniffing Head nodding	40 % 36 %	34 31	Falling after rearing Piano playing	56 % 55 %	53 52
Exploration w/sniffing	8 %	12	Immobility w/head movements	36 %	31	Chewing	44 %	42
Head nodding	7 %	10	Tonic head extension	33 %	28	Head nodding	43 %	41
Freezing	6 %	9	Arousal (”wake-up”) after seizure initiation	33 %	28	Tail extension	32 %	30
Sniffing	6 %	9	Freezing	22 %	19	Tonic head extension	31 %	29
Chewing	4 %	6	Left hind paw clonus	22 %	19	Immobility w/head movements	28 %	27
WDS	4 %	6	Right forepaw clonus	20 %	17	Unspecified body movement	22 %	21
Exploration	3 %	5	Arousal (”wake-up”) later during seizure	20 %	17	Left forepaw clonus	21 %	20
Clonic body jerks	3 %	5	Piano playing	19 %	16	Clonic body jerks	19 %	18
Rearing	3 %	4	Rearing	19 %	16	Exploration w/sniffing	19 %	18
Tail extension	3 %	4	Chewing	18 %	15	Right forepaw clonus	17 %	16
Head clonus	3 %	4	Unspecified body movement	7 %	6	Sniffing	17 %	16
Slow ”scanning” horizontal head movement	3 %	4	Head clonus	7 %	6	Arousal (”wake-up”) later during seizure	16 %	15
Left forepaw clonus	2 %	3	Exploration	7 %	6	WDS	14 %	13
Yawning	2 %	3	Tail extension	7 %	6	Left hind paw clonus	11 %	10
Slow wandering	2 %	3	Sniffing	6 %	5	Salivation	9 %	9
Left eye blinking	2 %	3	Clonic body jerks	6 %	5	Freezing	8 %	8
Left hind paw clonus	1 %	2	Rhythmic left whisker movement	6 %	5	Exploration	7 %	7
Head turning to right	1 %	2	Right hind paw clonus	5 %	4	Left eye blinking	7 %	7
Tonic body extension	1 %	1	Bilateral forepaw clonus	5 %	4	Slow ”scanning” horizontal head movement	4 %	4
Left ear clonus	1 %	1	Slow ”scanning” horizontal head movement	5 %	4	Head turning to right	4 %	4
Right eye blinking	1 %	1	Slow wandering	5 %	4	Fast breathing	4 %	4
Right forepaw clonus	1 %	1	Rhythmic bilateral whisker movements	5 %	4	Right hind paw clonus	3 %	3
Right hind paw clonus	1 %	1	Head turning to right	2 %	2	Bilateral forepaw clonus	3 %	3
Rhythmic bilateral whisker movements	1 %	1	Rhythmic right whisker movement	2 %	2	Right eye blinking	3 %	3
			Body turning right	2 %	2	Rhythmic left whisker movement	2 %	2
			Left eye blinking	2 %	2	Rhythmic bilateral whisker movements	2 %	2
			Left ear clonus	2 %	2	Body turning left	2 %	2
			Agitation	1 %	1	Head turning to left	2 %	2
			Fast breathing	1 %	1	Left ear clonus	2 %	2
			Freezing in rearing position	1 %	1	Body turning right	1 %	1
						Tonic body extension	1 %	1
						Body curling	1 %	1
Postictal phase			Postictal phase			Postictal phase		
Feature (9)	%	#Sz	Feature (8)	%	#Sz	Feature (9)	%	#Sz
Immobility	74 %	111	Immobility	61 %	52	Immobility	43 %	41
Immobility w/head movements	26 %	38	WDS	41 %	35	Exploration	36 %	34
Exploration	9 %	13	Immobility w/head movements	39 %	33	Immobility w/head movements	35 %	33
WDS	7 %	10	Exploration	27 %	23	WDS	29 %	28
Exploration w/sniffing	6 %	9	Chewing	4 %	3	Clonic body jerks	17 %	16
Unspecified body movement	5 %	8	Freezing	2 %	2	Slow ”scanning” horizontal head movement	9 %	9
Slow ”scanning” horizontal head movement	3 %	5	Slow wandering	1 %	1	Rearing	6 %	6
Freezing	1 %	1	Exploration w/sniffing	1 %	1	Unspecified body movement	1 %	1
Yawning	1 %	1				Chewing	1 %	1

***Abbreviations:*** #Sz, number of seizures with the feature; WDS, wet-dog shake;

1 arousal after seizure initiation refers to “wake-up” < 5 s after electrographic seizure initiation;

2 arousal later during seizure refers to “wake-up” > 5 s after electrographic seizure initiation.

## Data Availability

Data will be made available on request.

## Appendix A. Supporting information

Supplementary data associated with this article can be found in the online version at doi:10.1016/j.jneumeth.2025.110671.
