# Supplementary material for: Feature library for behavioural characterization of early and late seizures in an experimental model of post-traumatic epilepsy
Source: J Neurosci Methods. Author manuscript; Available in PMC 2026 Jul 21. (PMC13384811; doi:10.1016/j.jneumeth.2025.110671)
Supplement: 1 [file NIHMS2189850-supplement-1.docx]

**Supplementary Material**

Videos 1–10 demonstrate the pre-ictal, ictal, and post-ictal behavioral features described in the text. The videos were recorded in 2K resolution and synchronized with the electroencephalogram (EEG). The 4-channel EEG was used for seizure detection and for defining the beginning and end of the ictal phase in the EpiBioS4Rx Project 1 (Ndode-Ekane et al., 2024). Note that, due to journal requirements, the video resolution was reduced to 1080p. Only newly appearing features were annotated. Videos 5-10 do not show the entire ictal phase due to file size limitations. The colored bar indicates the ictal phase.

Video1 Sleep N3 → Arousal ("wake-up") after sz initiation → Immobility w/head movements → Chewing → Rearing → Piano playing → Falling after rearing → Right forepaw clonus → Tonic head extension → Tail extension → Immobility → Immobility w/head movements

Animal #1171

Late seizure

Duration 169 sec

Racine 5

Seizure onset: Sleep (N3)

Preictal-ictal transition: From sleep to arousal after seizure initiation

Ictal-postictal transition: From rearing to immobility

Video2 Immobility → Arousal ("wake-up") later during sz → Sniffing → Exploration w/sniffing → Freezing in Rearing → Chewing → R forepaw clonus → Rearing → Falling after rearing → L forepaw clonus → Piano playing → Salivation → Rhythmic bilat whisker → Exploration → Clonic body jerks

Animal #1071

Late seizure

Duration 146 sec

Racine 5

Seizure onset: Immobility (sleep stage could not be defined)

Preictal-ictal transition: From immobility to arousal after seizure initiation

Ictal-postictal transition: From rearing to exploration

Video3 Awake → Immobility → Slow "scanning" horizontal head movement → Exploration w/sniffing → Freezing → Head nodding → Right forepaw clonus → Immobility → Immobility → Immobility w/head movements

Animal #1149

Post-implantation seizure

Duration 106 sec

Racine 2

Seizure onset: Awake

Preictal-ictal transition: From awake to brief immobility to slow “scanning” horizontal head movement

Ictal-postictal transition: From immobility to immobility

Video4 NCSE → Immobility → Arousal ("wake-up") after SZ initialization → Unspecified body movement → Tail extension → Tonic body extension

#1177

Early seizure

Duration 88 sec

Racine 0

Seizure onset: Immobility (NCSE)

Preictal-ictal transition: From immobility to immobility

Ictal-postictal transition: From immobility to immobility (not shown)

Video5 Exploration w/sniffing → Freezing → Head clonus

#1152

Post-implantation seizure

Duration 130 sec

Racine 2

Video6 Exploration w/sniffing → Body curling → Fast breathing

#1140

Late seizure

Duration 58 sec

Racine 0

Video7 SlowWandering

#1194

Early seizure

Duration 99 sec

Racine 0

Video8 Body Turning Right

#1140

Post-implantation seizure

Duration 119 sec

Racine 4

Video9 Immobility w/head movements → Left hindpaw clonus→ Left forepaw clonus

#1194

Early seizure

Duration 118 sec

Racine 2

Video10 Exploration w/sniffing → Agitation → Immobility

Animal #1149

Post-implantation seizure

Duration 88 sec

Racine 0
