# Supplementary material for: Feature library for behavioural characterization of early and late seizures in an experimental model of post-traumatic epilepsy
Source: J Neurosci Methods. Author manuscript; Available in PMC 2026 Jul 21. (PMC13384811; doi:10.1016/j.jneumeth.2025.110671)
Supplement: 5 [file NIHMS2189850-supplement-5.docx]

| **Supplementary Materials (Tables S1-S5 and Figure S1)**  **Table S1.** Description of behavioural features (semiology) of the pre-ictal, ictal, and post-ictal phase of early, post-electrode implantation, and late seizures listed in **Table 1**. A total of 3 pre-ictal, 43 ictal, and 13 post-ictal behavioural features were annotated based on high-resolution videos that were time-locked with electroencephalogram (EEG). Pre-ictal phase was defined as the preceding 5 seconds of electrographic seizure onset. The postictal phase included a 30-second period immediately following the end of an electrographic seizure. Illustrative video-EEG examples are shown in **Video 2**. | | |
| --- | --- | --- |
| **Category** | **Feature** | **Description** |
| **Pre-ictal phase (3)** | | |
|  | In awake | Awake, based on EEG, while the animal appears fully conscious and alert |
|  | In immobility | In sleep, based on EEG |
|  | sleep | Sleep-wake stage in EEG undetermined, while the animal is immobile |
| **Ictal phase (43)** | | |
| Consciousness (7) |  | |
|  | Arousal (”wake-up”) after seizure  initiation^1^ | Animal appears alert immediately after the seizure onset |
|  | Arousal (”wake-up”) later during  seizure^2^ | Animal appears alert seconds after the seizure onset |
|  | Immobility | Animal does not show any movement or motion |
|  | Immobility w/head movements | Animal is in a state of stillness accompanied by occasional movement of the head |
|  | Exploration | Animal is investigating the environment, involving movement and sensory (e.g., sniffing) exploration |
|  | Exploration w/sniffing | Animal is exploring surroundings in a disoriented or uncertain manner, accompanied by active sniffing - almost like looking for something |
|  | Slow wandering | Animal’s movement is slow and meandering |
| Mouth and whiskers (5) |  | |
|  | Chewing | Animal is chewing with an empty mouth or with food/other substance in the mouth |
|  | Sniffing | Animal is sniffing the surroundings with normal appearing whisker movements |
|  | Rhythmic right whisker movement | Animal shows rhythmic (clonic) motion of the right whiskers |
|  | Rhythmic left whisker movement | Animal shows rhythmic (clonic) motion of the left whiskers |
|  | Rhythmic bilateral whisker  movements | Animal shows rhythmic (clonic) bilateral motion of the whiskers |
| Eyes (2) |  |  |
|  | Left eye blinking | Animal shows short rhythmic closing and opening of the left eye |
|  | Right eye blinking | Animal shows short rhythmic closing and opening of the right eye |
| Head (7) |  | |
|  | Head turning to left | Rotational movement of the animal’s head to the left |
|  | Head turning to right | Rotational movement of the animal’s head to the right |
|  | Slow ”scanning” horizontal head  movement | Slow horizontal movement of the animal’s head (from left to right or right to left), as if scanning |
|  | Head nodding | Repetitive slow and “smooth” up and down movement of animal’s head |
|  | Head clonus | Rapid rhythmic jerking of animal’s head |
|  | Tonic head extension | Prolonged upward or backward extension of animal’s head |
|  | Yawning | Yawning |
| Ears (2) |  | |
|  | Left ear clonus | Animal shows rhythmic (clonic) movement or twitching of the left ear |
|  | Right ear clonus | Animal shows rhythmic (clonic) movement or twitching of the right ear |
| Paws (6) |  |  |
|  | Left forepaw clonus | Animal shows rhythmic (clonic) movement or twitching of the left forepaw |
|  | Left hind paw clonus | Animal shows rhythmic (clonic) movement or twitching of the left hind paw |
|  | Bilateral forepaw clonus | Animal shows rhythmic (clonic) movement or twitching of both forepaws simultaneously |
|  | Piano playing | Animal shows rhythmic (clonic) tapping movement of the forepaws (and head) while in the rearing position |
|  | Right forepaw clonus | Animal shows rhythmic (clonic) movement or twitching of the right forepaw |
|  | Right hind paw clonus | Animal shows rhythmic (clonic) movement or twitching of the right hind paw |
| Body and tail (12) |  | |
|  | Freezing | Animal “freezes” in a particular position usually in the middle of a movement |
|  | Unspecified body movement | Animal shows nonspecific (often appearing clonic) movement of the entire body |
|  | Rearing | Animal is standing on its hind paws with front paws in air accompanied by body extension |
|  | Freezing in rearing position | Animal remains motionless while in a rearing position |
|  | Falling after rearing | Animal loses its balance and falls down after rearing |
|  | Agitation | Animal is restless and hyperactive |
|  | Tonic body extension | Animal shows sustained hyperextension of the body |
|  | Clonic body jerks | Animal shows rapid, repetitive muscle contractions and relaxations, resulting in jerking movements |
|  | Body turning left | Animal shows rotational movement of the body to left |
|  | Body turning right | Animal shows rotational movement of the body to right |
|  | Body curling | Animal shows curving or bending its body into a rounded or curled shape |
|  | Tail extension | Animal directs its straight and rigid tail away from the body |
| Autonomic (2) |  |  |
|  | Fast breathing | Animal’s respiratory rate accelerates |
|  | Salivation | Animal shows foam/saliva dripping from the mouth |
| WDS (1) |  | |
|  | WDS | Animal shows paroxysmal whole body shake during a seizure (wet-dog shakes) |
| **Post-ictal phase (13)** | | |
|  | Immobility | Animal does not show any movement or motion |
|  | Immobility w/head movements | Animal is in a state of stillness accompanied by occasional movement of the head |
|  | Slow wandering | Animal’s movement is slow and meandering |
|  | Slow ”scanning” horizontal head  movement | Slow horizontal movement of the animal’s head (from left to right or right to left), as if scanning |
|  | Exploration | Animal is investigating the environment, involving movement and sensory (e.g., sniffing) exploration |
|  | Rearing | Animal is standing on its hind paws with front paws in the air accompanied **by** body extension |
|  | Agitation | Animal shows increased activity/restlessness following a seizure |
|  | Chewing | Animal is chewing with empty mouth or with food/other substance in the mouth |
|  | Yawning | Yawning |
|  | Salivation | Animal shows foam/saliva dripping from the mouth |
|  | WDS | Animal shows paroxysmal whole body shake after the electrographic seizure has ended |
|  | Clonic body jerks | Animal shows rapid, repetitive muscle contractions and relaxations after the electrographic seizure has ended |
|  | Unspecified body movement | Animal shows nonspecific (often clonic) movement of the entire body |
| ***Abbreviations:*** WDS, wet-dog shake; ^1^arousal after seizure initiation refers to “wake-up” <5 seconds after electrographic seizure initiation; ^2^arousal later during seizure refers to “wake-up” >5 seconds after electrographic seizure initiation. Number of features is shown in parenthesis. | | |

| **Table S2.** Pre-ictal to ictal transition. Frequency of the first behavioral feature during the ictal phase of the 149 early, 85 post-implantation, and 95 late seizures. The last pre-ictal feature was annotated within the 5 seconds before the onset of the electrographic seizure. The first ictal feature was annotated within 5 seconds after the onset of the electrographic seizure. | | |
| --- | --- | --- |
| **Last pre-ictal**  **behavioural feature** | **First ictal behavioural feature** | **% of all** |
| **Pre-ictal to ictal transition - Early seizures (149)** | | |
| **In immobility** |  | **77% (114/149)** |
|  | Immobility | 67% (76/114) |
|  | Arousal (”wake-up” ) after seizure  initiation^1^ | 18% (20/114) |
|  | Arousal (”wake-up” ) later during seizure^2^ | 7% (8/114) |
|  | Immobility w/head movements | 3% (3/114) |
|  | Unspecified body movement | 3% (3/114) |
|  | Chewing | 1% (1/114) |
|  | Rhythmic bilateral whisker movements | 1% (1/114) |
|  | Freezing | 1% (1/114) |
|  | Head nodding | 1% (1/114) |
| **In awake** |  | **20% (30/149)** |
|  | Immobility w/head movements | 30% (9/30) |
|  | Immobility | 27% (8/30) |
|  | Unspecified body movement | 13% (4/30) |
|  | Freezing | 7% (2/30) |
|  | Exploration | 7% (2/30) |
|  | Arousal (”wake-up” ) after seizure  initiation | 3% (1/30) |
|  | Exploration w/sniffing | 3% (1/30) |
|  | WDS | 3% (1/30) |
|  | Slow ”scanning” horizontal head  movement | 3% (1/30) |
|  | Slow wandering | 3% (1/30) |
| **In sleep** |  | **3% (5/149)** |
|  | Arousal (”wake-up” ) after seizure  initiation | 80% (4/5) |
|  | Unspecified body movement | 20% (1/5) |
| **Pre-ictal to ictal transition - Post-implantation seizures (85)** | | |
| **In sleep** |  | **53% (45/85)** |
|  | Arousal (”wake-up” ) after seizure  initiation | 51% (23/45) |
|  | Immobility | 44% (20/45) |
|  | Arousal (”wake-up” ) later during seizure | 4% (2/45) |
| **In awake** |  | **27% (23/85)** |
|  | Immobility | 48% (11/23) |
|  | Immobility w/head movements | 22% (5/23) |
|  | Exploration w/sniffing | 13% (3/23) |
|  | Sniffing | 4% (1/23) |
|  | WDS | 4% (1/23) |
|  | Exploration | 4% (1/23) |
|  | Body turning right | 4% (1/23) |
| **In immobility** |  | **20% (17/85)** |
|  | Immobility | 65% (11/17) |
|  | Arousal (”wake-up” ) after seizure  initiation | 24% (4/17) |
|  | Arousal (”wake-up” ) later during seizure | 12% (2/17) |
| **Pre-ictal to ictal transition - Late seizures (95)** | | |
| **In sleep** |  | **57% (54/95)** |
|  | Arousal (”wake-up” ) after seizure  initiation | 70% (38/54) |
|  | Immobility | 30% (16/54) |
| **In immobility** |  | **39% (37/95)** |
|  | Arousal (”wake-up” ) after seizure  initiation | 65% (24/37) |
|  | Immobility | 32% (12/37) |
|  | Salivation | 3% (1/37) |
| **In awake** |  | **4% (4/95)** |
|  | Immobility w/head movements | 25% (1/4) |
|  | Immobility | 25% (1/4) |
|  | Exploration | 25% (1/4) |
|  | Exploration w/sniffing | 25% (1/4) |
| ***Abbreviations***: WDS, wet-dog shake; ^1^arousal after seizure initiation refers to “wake-up” <5 sec after electrographic seizure initiation; ^2^arousal later during seizure refers to “wake-up” >5 sec after electrographic seizure initiation. | | |

| **Table S3**. Frequency of the first behavioral feature during the ictal phase of the 149 early, 85 post-implantation, and 95 late seizures | | | |
| --- | --- | --- | --- |
| **1^st^ Ictal feature - Early seizures (149)** | | | |
| **Feature (14)** | **Percentage** | **Number of seizures** |  |
| Immobility | 56 % | 84/149 |  |
| Arousal (”wake-up”) after seizure initiation^1^ | 17 % | 25/149 |  |
| Immobility w/head movements | 8 % | 12/149 |  |
| Unspecified body movement | 5 % | 8/149 |  |
| Arousal (”wake-up”) later during seizure^2^ | 5 % | 8/149 |  |
| Freezing | 2 % | 3/149 |  |
| Exploration | 1 % | 2/149 |  |
| Slow ”scanning” horizontal head movement | 1 % | 1/149 |  |
| Exploration w/sniffing | 1 % | 1/149 |  |
| Slow wandering | 1 % | 1/149 |  |
| WDS | 1 % | 1/149 |  |
| Chewing | 1 % | 1/149 |  |
| Rhythmic bilateral whisker movements | 1 % | 1/149 |  |
| Head nodding | 1 % | 1/149 |  |
| **1^st^ Ictal feature - Post-implantation seizures (85)** | | | |
| **Feature (9)** | **Percentage** | **Number of seizures** |  |
| Immobility | 49 % | 42/85 |  |
| Arousal (”wake-up”) after seizure initiation | 32 % | 27/85 |  |
| Immobility w/head movements | 6 % | 5/85 |  |
| Arousal (”wake-up”) later during seizure | 5 % | 4/85 |  |
| Exploration w/sniffing | 4 % | 3/85 |  |
| WDS | 1 % | 1/85 |  |
| Sniffing | 1 % | 1/85 |  |
| Exploration | 1 % | 1/85 |  |
| Body turning right | 1 % | 1/85 |  |
| **1^st^ Ictal feature - Late seizures (95)** | | | |
| **Feature (6)** | **Percentage** | **Number of seizures** |  |
| Arousal (”wake-up”) after seizure initiation | 65 % | 62/95 |  |
| Immobility | 31 % | 29/95 |  |
| Immobility w/head movements | 1 % | 1/95 |  |
| Exploration | 1 % | 1/95 |  |
| Salivation | 1 % | 1/95 |  |
| Exploration w/sniffing | 1 % | 1/95 |  |
| ***Abbreviations:*** WDS, wet-dog shake; ^1^arousal after seizure initiation refers to “wake-up” <5 sec after electrographic seizure initiation; ^2^arousal later during seizure refers to “wake-up” >5 sec after electrographic seizure initiation. | | | |

| **Table S4.** Ictal to post-ictal transition. The last ictal and the first post-ictal behavioral feature of the early, post-implantation, and late seizures. | | |
| --- | --- | --- |
| **Last ictal feature during electrographic seizure** | **First post-ictal feature after the end of electrographic seizure** | **% of all seizures** |
| **Ictal to post-ictal transition - Early seizures (149)** | | |
| No behavioural change at the end of electrographic seizure 83% (123/149) | | |
| **Immobility** |  | **65% (97/149)** |
|  | Immobility | 90% (87/97) |
|  | Immobility w/head movements | 6% (6/97) |
|  | Exploration | 2% (2/97) |
|  | Unspecified body movement | 2% (2/97) |
| **Immobility w/head movements** |  | **13% (20/149)** |
|  | Immobility w/head movements | 90% (18/20) |
|  | Exploration | 5% (1/20) |
|  | WDS | 5% (1/20) |
| **Exploration w/sniffing** |  | **7% (10/149)** |
|  | Exploration w/sniffing | 100% (10/10) |
| **Exploration** |  | **3% (5/149)** |
|  | Exploration | 80% (4/5) |
|  | Immobility | 20% (1/5) |
| **WDS** |  | **3% (5/149)** |
|  | Immobility w/head movements | 40% (2/5) |
|  | Exploration | 40% (2/5) |
|  | WDS | 20% (1/5) |
| **Unspecified body movement** |  | **3% (4/149)** |
|  | Immobility | 50% (2/4) |
|  | Unspecified body movement | 50% (2/4) |
| **Slow ”scanning” horizontal head movement** |  | **1% (2/149)** |
|  | Immobility w/head movements | 50% (1/2) |
|  | Slow ”scanning” horizontal head  movement | 50% (1/2) |
| **Rearing** |  | **1% (2/149)** |
|  | Immobility | 100% (2/2) |
| **Clonic body jerks** |  | **1% (1/149)** |
|  | Unspecified body movement | 100% (1/1) |
| **Freezing** |  | **1% (1/149)** |
|  | Freezing | 100% (1/1) |
| **Slow wandering** |  | **1% (1/149)** |
|  | Immobility | 100% (1/1) |
| **Tonic body extension** |  | **1% (1/149)** |
|  | Exploration | 100% (1/1) |
| **Ictal to post-ictal transition - Post-implantation seizures (85)** | | |
| No behavioural change at the end of electrographic seizure 64% (54/85) | | |
| **Immobility** |  | **49% (42/85)** |
|  | Immobility | 76% (32/42) |
|  | Immobility w/head movements | 14% (6/42) |
|  | WDS | 5% (2/42) |
|  | Exploration | 5% (2/42) |
| **WDS** |  | **22% (19/85)** |
|  | WDS | 53% (10/19) |
|  | Immobility | 21% (4/19) |
|  | Immobility w/head movements | 16% (3/19) |
|  | Exploration | 11% (2/19) |
| **Immobility w/head movements** |  | **8% (7/85)** |
|  | Immobility w/head movements | 71% (5/7) |
|  | Exploration | 29% (2/7) |
| **Unspecified body movement** |  | **4% (3/85)** |
|  | Immobility | 100% (3/3) |
| **Exploration** |  | **4% (3/85)** |
|  | Exploration | 100% (3/3) |
| **Exploration w/sniffing** |  | **2% (2/85)** |
|  | Immobility | 50% (1/2) |
|  | Exploration w/sniffing | 50% (1/2) |
| **Freezing** |  | **2% (2/85)** |
|  | Freezing | 100% (2/2) |
| **Left forepaw clonus** |  | **2% (2/85)** |
|  | Immobility | 100% (2/2) |
| **Head clonus** |  | **1% (1/85)** |
|  | Immobility | 100% (1/1) |
| **Tonic head extension** |  | **1% (1/85)** |
|  | Immobility | 100% (1/1) |
| **Head nodding** |  | **1% (1/85)** |
|  | Immobility | 100% (1/1) |
| **Slow wandering** |  | **1% (1/85)** |
|  | Slow wandering | 100% (1/1) |
| **Chewing** |  | **1% (1/85)** |
|  | WDS | 100% (1/1) |
| **Ictal to post-ictal transition - Late seizures (95)** | | |
| No behavioural change at the end of electrographic seizure 57% (54/95) | | |
| **Immobility** |  | **41% (39/95)** |
|  | Immobility | 62% (24/39) |
|  | Immobility w/head movements | 15% (6/39) |
|  | WDS | 13% (5/39) |
|  | Exploration | 5% (2/39) |
|  | Unspecified body movement | 5% (2/39) |
| **Rearing** |  | **19% (18/95)** |
|  | Rearing | 33% (6/18) |
|  | Immobility | 28% (5/18) |
|  | Exploration | 17% (3/18) |
|  | Immobility w/head movements | 17% (3/18) |
|  | WDS | 6% (1/18) |
| **Immobility w/head**  **movements** |  | **14% (13/95)** |
|  | Immobility w/head movements | 92% (12/13) |
|  | Exploration | 8% (1/13) |
| **Clonic body jerks** |  | **5% (5/95)** |
|  | Clonic body jerks | 40% (2/5) |
|  | Immobility w/head movements | 40% (2/5) |
|  | Immobility | 20% (1/5) |
| **Exploration** |  | **5% (5/95)** |
|  | Exploration | 100% (5/5) |
| **WDS** |  | **5% (5/95)** |
|  | Immobility w/head movements | 40% (2/5) |
|  | WDS | 20% (1/5) |
|  | Exploration | 20% (1/5) |
|  | Immobility | 20% (1/5) |
| **Slow ”scanning”**  **horizontal head**  **movement** |  | **4% (4/95)** |
|  | Slow ”scanning” horizontal head  movement | 100% (4/4) |
| **Unspecified body**  **movement** |  | **4% (4/95)** |
|  | Immobility | 75% (3/4) |
|  | WDS | 25% (1/4) |
| **Piano playing** |  | **1% (1/95)** |
|  | Immobility | 100% (1/1) |
| **Head nodding** |  | **1% (1/95)** |
|  | WDS | 100% (1/1) |
| ***Abbreviations:*** WDS, wet-dog shake. | | |

| **Table S5**. Type and frequency of the first behavioral feature during the post-ictal phase of the early, post-implantation, and late seizures. | |
| --- | --- |
| **1^st^ Postictal feature - Early seizures (149)** | |
| Feature | Percentage of all seizures |
| Immobility | 62% (93/149) |
| Immobility w/head movements | 18% (27/149) |
| Exploration | 7% (10/149) |
| Exploration w/sniffing | 7% (10/149) |
| Unspecified body movement | 3% (5/149) |
| WDS | 1% (2/149) |
| Freezing | 1% (1/149) |
| Slow ”scanning” horizontal head movement | 1% (1/149) |
| **1^st^ Postictal feature - Post-implantation seizures (85)** | |
| Immobility | 53% (45/85) |
| Immobility w/head movements | 16% (14/85) |
| WDS | 15% (13/85) |
| Exploration | 11% (9/85) |
| Freezing | 2% (2/85) |
| Exploration w/sniffing | 1% (1/85) |
| Slow wandering | 1% (1/85) |
| **1^st^ Postictal feature - Late seizures (95)** | |
| Immobility | 37% (35/95) |
| Immobility w/head movements | 26% (25/95) |
| Exploration | 13% (12/95) |
| WDS | 9% (9/95) |
| Rearing | 6% (6/95) |
| Slow ”scanning” horizontal head movement | 4% (4/95) |
| Clonic body jerks | 2% (2/95) |
| Unspecified body movement | 2% (2/95) |
| ***Abbreviation:*** WDS, wet-dog shake. | |

**
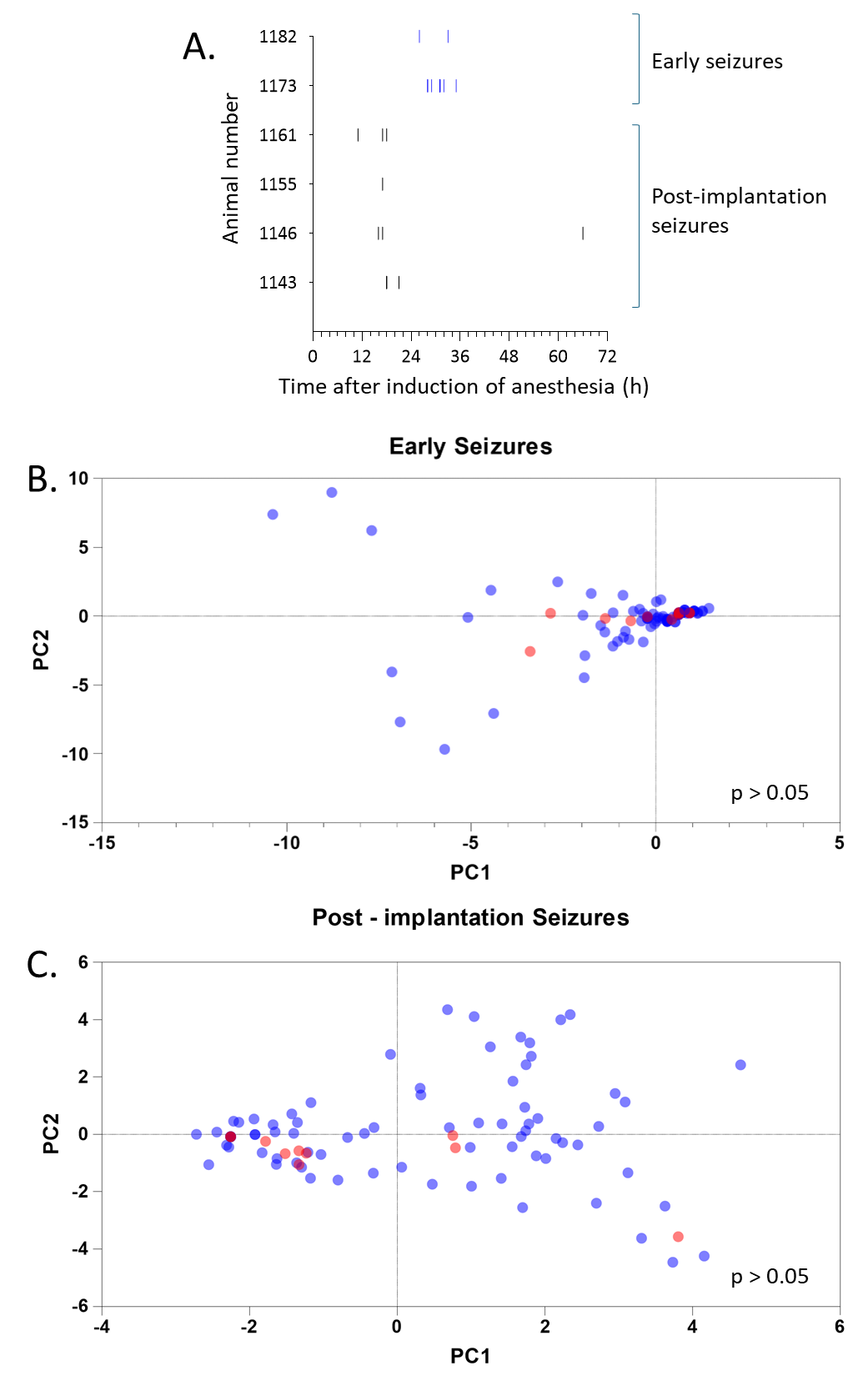
**

**Figure S1.** ***Early and post-implantation seizures in sham-operated rats.*** Sham-operated animals included in this preliminary analysis underwent the same procedures (craniotomy, electrode-implantation) as TBI animals but were not exposed to lateral fluid-percussion-induced impact. **(A) *Raster plot*** ***showing the occurrence of early and post-implantation seizures in sham-operated rats.*** ***Early seizures*** - The 11 early seizures analysed occurred in 2 sham-operated rats (EEG cohort; animals #1173, #1182; analysis D0-D7 after sham-operation) with a total of 61 annotated behavioural features. The average delay from the initiation of anaesthesia to seizure occurrence was 30.5 + 2.4 h (median 31 h, range 26-35 h). The average number of features per seizure was 5.5 ± 1.7 (median 6, range 3-9). The average duration of the ictal phase was 34 ± 14 s (median 37 s, range 11-59 s). The average Racine score was 0.4+ 0.6 (median 0, range 0-2). ***Post-implantation seizures*** - The 149 post-implantation seizures analysed occurred in 4 sham-operated rats (MRI cohort; animals #1143, # 1146, #1155, #1161; 6^th^ post-TBI month) with a total of 100 annotated behavioural features. The average delay from the initiation of anaesthesia to seizure occurrence was 21.9 + 14.9 h (median 17.5 h, range 11-66 h). The average number of features per seizure was 10 + 5.1 (median 9, range 3-16). The average duration of the ictal phase was 79 + 37 s (median 79.5 s, range 20-129 s). The average Racine score was 0.9 + 1.1 (median 0, range 0-3). **(B-C)** ***PCA of behavioral features and Racine scale in sham-operated and TBI rats*.** Principal component analysis did not reveal any separation of behavioral seizure phenotypes between sham (red color dots) and TBI (blue color) rats in **(B)** early or **(C)** post-implantation seizures. Each dot refers to one seizure, with the location in the graph determined by the first two principal components (PC1 and PC2).
